# Supplementary material for: The breadth of the Mexican Transition Zone as defined by its flowering plant generic flora
Source: PLoS One. 2020 Jun 25;15(6):e0235267. doi: 10.1371/journal.pone.0235267 (PMC7316278; doi:10.1371/journal.pone.0235267)
Supplement: S1 Data — (DOCX) [file pone.0235267.s001.docx]

Appendix 1. Distinctive genera of Mexico (with half or more of their species registered in the country) and the biogeographic provinces where they are distributed. The number above the diagonal indicates the total number of species in the genus and below the diagonal the number of species in Mexico. In brackets their presence is indicated in the three main biogeographic regions shown in Figure 2: NEA = Nearctic Region, NEO = Neotropical Region, MTZ = Mexican Transition Zone. Abbreviations of the biogeographical provinces: ALCH= Altos de Chiapas, ALT= Altiplano Norte (Chihuahuense), ALS= Altiplano Sur (Zacatecano-Potosino), BAL= Cuenca del Balsas, BCA= Baja California, CABO= Del Cabo, CAL= California, CGM= Costa del Golfo, CPA= Costa del Pacífico, EVT= Eje Volcánico Transversal, OAX= Oaxaca, PET= Petén, SMOC= Sierra Madre Occidental, SMOR= Sierra Madre Oriental, SMS= Sierra Madre del Sur, SOC= Soconusco, SON= Sonora, TAM= Tamaulipas, YUC= Yucatán. An asterisk indicates the genera endemic to Mexico.

**Acanthaceae**

*Anisacanthus* Nees, 1842. 12/10. ALTN, ALTS, BAL, CGM, CPA, EVT, OAX, PET, SMOC, SMOR, SMS, SON, YUC. [NEA, MTZ].

**Aphanosperma* T.F. Daniel, 1988. 1/1. BCA, CABO, CPA, SON. [NEA, MTZ].

*Beloperone* Nees, 1832. 2/2. ALTS, EVT, SMOR. [NEO, MTZ].

*Bravaisia* DC., 1838. 3/3. ALTS, BAL, CGM, CPA, OAX, PET, SOC, YUC. [NEO, MTZ].

**Buceragenia* Greenm., 1897. 2/2. BAL. [NEA, MTZ].

*Carlowrightia* A. Gray, 1878. 24/24. ALCH, ALTN, ALTS, BAL, BCA, CABO, CGM, CPA, EVT, OAX, PET, SMOC, SMOR, SMS, SOC, SON, TAM, YUC. [MTZ].

**Chalarothyrsus* Lindau, 1904. 1/1. BAL, CPA, EVT, OAX, SMS. [NEA, MTZ].

*Chileranthemum* Oerst., 1855. 3/3. CGM, CPA, EVT, OAX, SMOR, SMS. [NEO, MTZ].

**Glockeria* Nees, 1847. 1/1. SMS. [NEA].

**Gypsacanthus* Lott, Jaramillo & Rzed., 1986. 1/1. BAL, CPA, OAX. [NEA, MTZ].

*Henrya* Nees ex Benth., 1845. 3/3. ALCH, ALTS, BAL, BCA, CABO, CGM, CPA, EVT, OAX, PET, SMOC, SMOR, SMS, SOC, YUC. [NEA, MTZ].

**Holographis* Nees, 1847. 18/18. ALCH, ALTN, ALTS, BAL, BCA, CABO, CGM, CPA, EVT, OAX, PET, SMOC, SMOR, SMS, SOC, SON, YUC. [NEA, MTZ].

**Hoverdenia* Nees, 1847. 1/1. ALTS, SMOR. [NEA, MTZ].

**Jacobinia* Moric., 1846. 5/5. ALTN, ALTS, BAL, CGM, CPA, EVT, OAX, SMOC, SMOR, SMS, TAM. [NEA, MTZ].

*Louteridium* S. Watson, 1888. 11/10. ALCH, ALTS, BAL, CGM, CPA, EVT, OAX, PET, SMOC, SMOR, SOC. [NEA, MTZ].

**Mexacanthus* T.F. Daniel, 1981. 1/1. CPA. [NEA].

**Mirandea* Rzed., 1959. 6/6. ALCH, ALTN, ALTS, CGM, SMOR, SOC. [NEA, MTZ].

*Poikilacanthus* Lindau, 1893. 8/5. ALCH, BAL, CGM, CPA, SMS, SOC. [NEO, MTZ].

*Spathacanthus* Baill., 1891. 4/2. ALCH, CGM, CPA, OAX, SMOR, SOC. [NEO, MTZ].

*Tetramerium* Nees, 1846. 29/23. ALCH, ALTN, ALTS, BAL, BCA, CABO, CGM, CPA, EVT, OAX, PET, SMOC, SMOR, SMS, SOC, SON, TAM, YUC. [MTZ].

*Yeatesia* Small, 1896. 2/2. ALTN, ALTS, SMOR. [NEA, MTZ].

**Achariaceae**

**Chiangiodendron* T. Wendt, 1988. 1/1. ALCH, CGM, SOC. [NEA].

**Achatocarpaceae**

*Achatocarpus* Triana, 1858. 5/4. ALTN, ALTS, BAL, CGM, CPA, OAX, PET, SOC, SON, TAM, YUC. [NEO, MTZ].

*Phaulothamnus* A. Gray, 1885. 1/1. ALTN, BCA, CABO, CPA, OAX, SON, TAM. [NEA, MTZ].

**Alismataceae**

*Echinodorus* Rich. ex Engelm., 1848. 9/9. ALCH, ALTN, ALTS, BAL, BCA, CABO, CAL, CGM, CPA, EVT, OAX, PET, SMOR, SOC, SON, TAM, YUC. [MTZ].

*Limnocharis* Humb. & Bonpl., 1807. 2/2. CGM, CPA, OAX, PET, SOC, YUC. [MTZ].

**Amaranthaceae**

*Aphanisma* Nutt. ex Moq., 1849. 1/1. BCA, CAL. [NEA].

*Chamissoa* Kunth, 1817. 3/3. ALCH, ALTS, BAL, CGM, CPA, EVT, OAX, PET, SMOR, SMS, SOC, YUC. [NEO, MTZ].

*Cycloloma* Moq., 1840. 1/1. ALTN. [NEA].

*Extriplex* E.H. Zacharias, 2010. 2/1. BCA, CAL. [NEA].

*Iresine* P. Browne, 1756. 50/33. ALCH, ALTN, ALTS, BAL, BCA, CABO, CGM, CPA, EVT, OAX, PET, SMOC, SMOR, SMS, SOC, SON, TAM, YUC. [MTZ].

**Lagrezia* Moq., 1849. 1/1. BAL, CGM, CPA, SOC. [NEA].

**Meiomeria* Standl., 1916. 1/1. ALTN, ALTS. [NEA].

*Sarcobatus* Nees, 1841. 2/1. SON. [NEA].

*Tidestromia* Standl., 1916. 9/9. ALTN, ALTS, BCA, CAL, CGM, CPA, SON, TAM. [NEA, MTZ].

**Amaryllidaceae**

**Diphalangium* Schauer, 1847. 1/1. [NEA].

*Hymenocallis* Salisb., 1812. 50/33. ALCH, ALTN, ALTS, BAL, CGM, CPA, EVT, OAX, PET, SMOC, SMS, SOC, SON, YUC. [MTZ].

**Sprekelia* Heist., 1755. 2/2. ALCH, ALTS, BAL, CGM, CPA, EVT, OAX, SMOC, SMOR, SMS, SOC. [NEA, MTZ].

**Anacampserotaceae**

*Grahamia* Gillies ex Hook. & Arn., 1833. 2/2. ALTN, ALTS. [NEA, MTZ].

**Talinaria* Brandegee, 1908. 2/2. ALTN, ALTS. [NEA].

**Anacardiaceae**

**Actinocheita* F.A. Barkley, 1937. 1/1. BAL, CGM, CPA, EVT, OAX, SMS. [NEA, MTZ].

*Amphipterygium* Schiede, 1843. 5/4. ALCH, ALTS, BAL, CGM, CPA, EVT, OAX, SMOC, SMOR, SMS, SOC. [NEO, MTZ].

*Attilaea* E. Martínez & Ramos, 2007. 1/1. PET, YUC. [NEA].

**Bonetiella* Rzed., 1957. 1/1. ALTN, ALTS, EVT, SMOR. [NEA, MTZ].

*Cyrtocarpa* Kunth, 1825. 5/4. ALCH, ALTS, BAL, BCA, CABO, CGM, CPA, EVT, OAX, SMOC, SMS, SOC. [NEO, MTZ].

*Malosma* Nutt. Ex Abrams, 1917. 1/1. BCA, CABO, CAL. [NEA].

*Mosquitoxylum* Krug & Urb., 1895. 1/1. ALCH, CGM, CPA, OAX, PET, SOC, YUC. [NEO].

**Pachycormus* Coville, 1911. 3/3. BCA. [NEA].

**Pseudosmodingium* Engl., 1881. 5/5. ALTS, BAL, CGM, CPA, EVT, OAX, PET, SMOC, SMOR, SMS, SOC, YUC. [NEA, MTZ].

*Rhus* L., 1753. 53/40. ALCH, ALTN, ALTS, BAL, BCA, CABO, CAL, CGM, CPA, EVT, OAX, SMOC, SMOR, SMS, SOC, SON, TAM. [MTZ].

**Annonaceae**

*Sapranthus* Seem., 1866. 8/4. ALCH, BAL, CGM, CPA, OAX, PET, SMOC, SOC, YUC. [NEO, MTZ].

**Tridimeris* Baill., 1869. 2/2. ALCH, CGM, CPA, OAX, SMOR, SOC. [NEA, MTZ].

**Apiaceae**

*Ammoselinum* Torr. & A. Gray, 1855. 4/2. ALTN, TAM. [NEA].

*Apiastrum* Nutt. ex Torr. & A. Gray, 1840. 1/1. BCA, CAL. [NEA].

*Arracacia* Bancr., 1828. 42/27. ALCH, ALTN, ALTS, BAL, BCA, CABO, CGM, CPA, EVT, OAX, SMOC, SMOR, SMS, SOC, TAM. [NEO, MTZ].

*Coaxana* J.M. Coult. & Rose, 1895. 2/2. BAL, CPA, OAX, SMS, SOC. [NEO, MTZ].

**Coulterophytum* B.L. Rob., 1892. 3/3. ALTS, BAL, CPA, EVT, SMOC. [NEA, MTZ].

**Dahliaphyllum* Constance & Breedlove, 1994. 1/1. SMS. [NEA].

*Donnellsmithia* J.M. Coult. & Rose, 1890. 20/20. ALCH, ALTN, ALTS, BAL, CPA, EVT, OAX, SMOC, SMOR, SMS, SOC. [NEO, MTZ].

*Enantiophylla* J.M. Coult. & Rose, 1893. 1/1. BAL, CPA, EVT, SMOC, SMOR, SMS. [NEO, MTZ].

**Mathiasella* Constance & C.L. Hitchc., 1954. 1/1. SMOR, TAM. [NEA, MTZ].

*Micropleura* Lag., 1825. 2/1. ALCH, BAL, CPA, EVT, OAX, SMOC, SMOR, SMS, SOC. [NEO, MTZ].

**Neogoezia* Hemsl., 1894. 4/4. BAL, EVT, OAX, SMOC, SMS. [NEA, MTZ].

*Neonelsonia* J.M. Coult. & Rose, 1895. 1/1. ALCH, CGM, SOC. [NEO].

*Ottoa* Kunth, 1821. 1/1. EVT, OAX, SMOR, SMS, SOC. [NEO, MTZ].

*Prionosciadium* S. Watson, 1888. 23/23. ALCH, ALTN, ALTS, BAL, CGM, CPA, EVT, OAX, SMOC, SMOR, SMS, SOC, SON. [NEA, MTZ].

*Rhodosciadium* S. Watson, 1889. 15/15. ALCH, ALTS, BAL, CPA, EVT, OAX, SMOC, SMOR, SMS, SOC. [NEO, MTZ].

*Spananthe* Jacq., 1791. 2/1. ALCH, BAL, CGM, CPA, EVT, OAX, SMOC, SMOR, SMS, SOC. [NEO, MTZ].

*Tauschia* Schltdl., 1835. 30/24. ALCH, ALTN, ALTS, CAL, EVT, OAX, SMOC, SMOR, SOC. [MTZ].

**Villarrealia* G.L. Nesom, 2012. 1/1. ALTN. [NEA].

*Yabea* Koso-Pol., 1915. 1/1. CAL, SON. [NEA].

**Apocynaceae**

*Allotoonia* J.F. Morales & J.K. Williams, 2004. 6/4. ALCH, BAL, CGM, CPA, PET, SMOR, SOC, YUC. [NEO, MTZ].

*Apocynum* L., 1753. 4/4. ALTN, ALTS, CAL, SMOC, SMOR, SON, TAM. [MTZ].

*Bruceholstia* Morillo, 2015. 1/1. ALCH, CGM, CPA, PET, SOC. [NEA, MTZ].

*Cascabela* Raf., 1838. 6/6. ALCH, ALTN, ALTS, BAL, CGM, CPA, EVT, OAX, PET, SMOC, SMOR, SMS, SOC, YUC. [MTZ].

*Dictyanthus* Decne., 1844. 16/16. ALCH, ALTN, ALTS, BAL, CGM, CPA, EVT, OAX, PET, SMOC, SMOR, SMS, SOC, SON, YUC. [NEA, MTZ].

*Funastrum* Fourn., 1882. 17/12. ALCH, ALTN, ALTS, BAL, BCA, CABO, CAL, CGM, CPA, EVT, OAX, PET, SMOC, SMOR, SMS, SOC, SON, TAM, YUC. [MTZ].

*Haplophyton* A. DC., 1844. 2/2. ALTN, BAL, CGM, CPA, OAX, SOC, SON. [MTZ].

**Microdactylon* Brandegee, 1908. 1/1. OAX. [NEA, MTZ].

*Pentalinon* Voigt, 1845. 2/1. ALCH, ALTS, BAL, CGM, CPA, EVT, OAX, PET, SMOR, SOC, YUC. [MTZ].

*Pherotrichis* Decne., 1838. 5/4. OAX, SMOC, SMS. [NEA, MTZ].

*Polystemma* Decne., 1844. 4/4. ALCH, ALTS, BAL, CGM, CPA, EVT, OAX, SMOC, SMOR, SMS, SOC. [MTZ].

*Prosthecidiscus* Donn. Sm., 1898. 1/1. BAL, CGM, CPA, OAX, SOC. [NEO, MTZ].

*Rhabdadenia* Müll. Arg., 1860. 3/2. CGM, CPA, OAX, PET, SOC, TAM, YUC. [MTZ].

*Rotundanthus* Morillo, 2015. 1/1. ALCH, CGM. [NEA].

*Seutera* Rchb., 1828. 4/3. ALCH, ALTN, ALTS, BAL, BCA, CABO, CAL, CGM, CPA, EVT, OAX, PET, SMOC, SMOR, SOC, SON, YUC. [MTZ].

**Suberogerens* Morillo, 2015. 1/1. BAL, CGM, CPA, OAX, SOC. [NEA, MTZ].

*Thenardia* Kunth, 1819. 3/3. ALCH, ALTS, BAL, CGM, CPA, EVT, OAX, SMOC, SMS, SOC. [NEA, MTZ].

*Thevetia* Adans., 1763. 3/3. ALCH, BAL, CGM, CPA, EVT, OAX, PET, SMOC, SMOR, SMS, SOC, YUC. [NEO, MTZ].

**Thoreauea* J.K. Williams, 2002. 3/3. OAX, SMS. [NEA, MTZ].

*Tintinnabularia* Woodson, 1936. 3/2. ALCH, CGM. [NEO].

*Trichosacme* Zucc., 1846. 1/1. ALTS. [NEA, MTZ].

*Vallesia* Ruiz & Pav., 1799. 12/7. ALCH, ALTN, ALTS, BAL, BCA, CABO, CGM, CPA, EVT, OAX, PET, SMOC, SMOR, SMS, SOC, SON, TAM, YUC. [MTZ].

*Vulcanoa* Morillo, 2015. 1/1. SOC. [NEA].

**Apodanthaceae**

*Apodanthes* Poit., 1824. 1/1. ALTN, ALTS. [NEO, MTZ].

**Araceae**

*Lemna* L., 1753. 14/7. ALCH, ALTN, ALTS, BAL, BCA, CABO, CAL, CGM, CPA, EVT, OAX, PET, SMOC, SMOR, SMS, SOC, SON, YUC. [MTZ].

*Pistia* L., 1753. 1/1. ALTS, BAL, CGM, CPA, OAX, PET, SOC, YUC. [MTZ].

*Spirodela* Schleid., 1839. 4/2. ALTN, ALTS, BAL, CGM, CPA. [MTZ].

**Araliaceae**

*Sciadodendron* Griseb., 1858. 2/1. CPA. [NEO, MTZ].

**Arecaceae**

*Acoelorrhaphe* Hook. f., 1883. 1/1. CGM, CPA, PET, SOC, YUC. [NEO].

*Brahea* Mart., 1836. 13/13. ALCH, ALTN, ALTS, BAL, BCA, CABO, CAL, CGM, CPA, EVT, OAX, SMOC, SMOR, SMS, SOC, SON. [NEA, MTZ].

*Chamaedorea* Willd., 1806. 104/53. ALCH, ALTS, BAL, CGM, CPA, EVT, OAX, PET, SMOC, SMOR, SMS, SOC, YUC. [MTZ].

*Reinhardtia* Liebm., 1836. 6/4. ALCH, CGM, OAX, PET, SOC. [NEO, MTZ].

*Sabal* Adans. ex Guers., 1804. 14/7. ALCH, ALTN, ALTS, BAL, BCA, CABO, CGM, CPA, EVT, OAX, PET, SMOC, SMOR, SOC, SON, TAM, YUC. [MTZ].

*Schippia* Burret, 1933. 1/1. CGM, PET. [NEO].

*Washingtonia* H. Wendl., 1879. 2/2. ALTN, BCA, CABO, CAL, CPA, SON. [NEA, MTZ].

**Asparagaceae**

*Agave* L., 1753. 208/195. ALCH, ALTN, ALTS, BAL, BCA, CABO, CAL, CGM, CPA, EVT, OAX, PET, SMOC, SMOR, SMS, SOC, SON, TAM, YUC. [MTZ].

*Beaucarnea* Lem., 1861. 13/13. ALCH, ALTN, ALTS, BAL, CGM, CPA, EVT, OAX, PET, SMOC, SMOR, SMS, SOC, YUC. [NEA, MTZ].

*Beschorneria* Kunth, 1850. 8/8. ALCH, ALTS, EVT, OAX, SMOR, SMS, SOC. [NEO, MTZ].

**Bessera* Schult. f., 1829. 3/3. ALTS, BAL, BCA, CABO, CPA, EVT, SMOC, SMS. [NEA, MTZ].

*Bloomeria* Kellogg, 1863. 3/2. CAL. [NEA].

**Dandya* H.E. Moore, 1953. 4/4. ALTN, ALTS, BAL, CPA. [NEA, MTZ].

*Dasylirion* Zucc., 1838. 24/24. ALTN, ALTS, BAL, CGM, CPA, EVT, OAX, SMOC, SMOR, SMS, SON, TAM. [NEA, MTZ].

*Furcraea* Vent., 1793. 23/15. ALCH, BAL, CGM, CPA, EVT, OAX, PET, SMOC, SMOR, SMS, SOC, YUC. [NEO, MTZ].

**Hemiphylacus* S. Watson, 1883. 5/5. ALTN, ALTS, OAX, SMOR. [NEA, MTZ].

*Hesperaloe* Engelm., 1871. 8/7. ALTN, ALTS, SMOC, SMOR, SON. [NEA, MTZ].

*Hesperocallis* A. Gray, 1843. 1/1. BCA, CAL, SON. [NEA].

*Hesperoyucca* (Engelm.) Baker, 1892. 3/2. BCA, CABO, CAL, SON. [NEA].

**Jaimehintonia* B.L. Turner, 1993. 1/1. SMOR. [NEA, MTZ].

*Manfreda* Salisb., 1866. 35/34. ALCH, ALTN, ALTS, BAL, CGM, CPA, EVT, OAX, PET, SMOC, SMOR, SMS, SOC, TAM, YUC. [NEA, MTZ].

*Milla* Cav., 1793. 11/11. ALCH, ALTN, ALTS, BAL, CABO, CGM, CPA, EVT, OAX, SMOC, SMOR, SMS, SOC, SON. [NEA, MTZ].

*Nolina* Michx., 1803. 30/23. ALCH, ALTN, ALTS, BAL, BCA, CABO, CAL, CPA, EVT, OAX, SMOC, SMOR, SMS, SOC, SON. [NEA, MTZ].

**Petronymphe* H.E. Moore, 1951. 2/2. BAL, SMS. [NEA].

**Prochnyanthes* Schmidel, 1763. 1/1. ALTS, EVT, SMOC, SMS. [NEA, MTZ].

*Triteleiopsis* Hoover, 1941. 1/1. BCA, SON. [NEA].

*Yucca* L., 1753. 49/34. ALCH, ALTN, ALTS, BAL, BCA, CABO, CAL, CGM, CPA, EVT, OAX, PET, SMOC, SMOR, SMS, SOC, SON, TAM, YUC. [MTZ].

**Asteraceae**

*Achyrachaena* Schauer, 1838. 1/1. CAL. [NEA].

*Achyropappus* Kunth, 1818. 3/2. ALTS, EVT, SMOR. [NEO, MTZ].

*Adenophyllum* Pers., 1807. 11/11. ALCH, ALTN, ALTS, BAL, BCA, CABO, CAL, CGM, CPA, EVT, OAX, SMOC, SMOR, SMS, SOC, SON, YUC. [MTZ].

**Adenothamnus* D.D. Keck, 1935. 1/1. BCA. [NEA].

**Ageratella* A. Gray ex S. Watson, 1887. 3/3. ALTS, EVT, SMOC. [NEA, MTZ].

*Ageratina* Spach, 1841. 322/169. ALCH, ALTN, ALTS, BAL, BCA, CABO, CAL, CGM, CPA, EVT, OAX, PET, SMOC, SMOR, SMS, SOC, SON, TAM. [MTZ].

*Alepidocline* S.F. Blake, 1934. 6/5. CPA, OAX, SMS, SOC. [NEO, MTZ].

*Alloispermum* Willd., 1807. 15/10. ALCH, ALTS, BAL, CGM, CPA, EVT, OAX, PET, SMOC, SMOR, SMS, SOC. [NEO, MTZ].

*Almutaster* Á. Löve & D. Löve, 1982. 1/1. ALTN, ALTS, EVT, OAX, SMOC. [NEA, MTZ].

*Alomia* Kunth, 1818. 4/4. ALTS, BAL, CPA, EVT, SMOR, SMS. [NEA, MTZ].

**Amauria* Benth., 1844. 3/3. BCA, CABO. [NEA].

*Amauriopsis* Rydb., 1914. 6/5. ALTN, ALTS, BCA, SON, TAM. [NEA, MTZ].

*Amblyolepis* DC., 1836. 1/1. ALTN, ALTS. [NEA].

*Amblyopappus* Hook. & Arn., 1841. 1/1. BCA, CAL. [NEA].

*Ambrosia* L., 1753. 40/29. ALCH, ALTN, ALTS, BAL, BCA, CABO, CAL, CGM, CPA, EVT, OAX, PET, SMOC, SMOR, SOC, SON, TAM, YUC. [MTZ].

*Amolinia* R.M. King & H. Rob., 1972. 1/1. CPA, SOC. [NEO].

*Ancistrocarphus* A. Gray, 1868. 2/1. CAL. [NEA].

*Anisocoma* Torr. & A. Gray, 1844. 1/1. CAL. [NEA].

**Aquilula* Nesom, 2018. 1/1. SMOR. [NEA, MTZ].

*Archibaccharis* Heering, 1904. 35/29. ALCH, ALTN, ALTS, BAL, BCA, CGM, CPA, EVT, OAX, PET, SMOC, SMOR, SMS, SOC. [NEO, MTZ].

**Arnicastrum* Greenm., 1903. 2/2. SMOC, SMS. [NEA, MTZ].

*Asanthus* R.M. King & H. Rob., 1972. 3/3. ALTN, ALTS, EVT, OAX, SMOC, SMOR, SON. [NEA, MTZ].

*Astranthium* Nutt., 1840. 12/9. ALCH, ALTN, ALTS, EVT, OAX, SMOC, SMOR, SMS, TAM. [NEA, MTZ].

*Atrichoseris* A. Gray, 1884. 1/1. CAL. [NEA].

**Axiniphyllum* Benth., 1872. 5/5. OAX, SMOC, SMS. [NEA, MTZ].

**Aztecaster* G.L. Nesom, 1993. 2/2. ALTN, ALTS, BAL, CPA, OAX, SMOR, SMS. [NEA, MTZ].

**Baeriopsis* J.T. Howell, 1942. 1/1. CAL. [NEA].

*Bahiopsis* Kellogg, 1863. 12/11. BCA, CABO, CAL, CPA, SMOC, SON. [NEA, MTZ].

*Baileya* Harv. & A. Gray ex A. Gray, 1849. 3/3. ALTN, ALTS, BCA, CAL, SMOC, SMOR, SON. [NEA, MTZ].

**Bajacalia* Loockerman, B.L. Turner & R.K. Jansen, 2003. 3/3. BCA, CABO, SON. [NEA].

*Baltimora* L., 1771. 2/2. ALCH, BAL, CGM, CPA, EVT, OAX, PET, SMOC, SMOR, SMS, SOC, YUC. [NEO, MTZ].

*Barkleyanthus* H. Rob. & Brettell, 1974. 1/1. ALCH, ALTN, ALTS, BAL, CGM, CPA, EVT, OAX, SMOC, SMOR, SMS, SOC, SON, TAM. [MTZ].

*Bartlettia* A. Gray, 1855. 1/1. ALTN, ALTS. [NEA].

*Bartlettina* R.M. King & H. Rob., 1971. 44/23. ALCH, BAL, CGM, CPA, EVT, OAX, PET, SMOC, SMOR, SMS, SOC. [NEO, MTZ].

**Batopilasia* G.L. Nesom & Noyes, 2000. 1/1. SMOC. [NEA, MTZ].

*Bebbia* Greene, 1885. 2/2. ALTN, BCA, CABO, CAL, CPA, SON. [NEA, MTZ].

*Bidens* L., 1753. 150/81. ALCH, ALTN, ALTS, BAL, BCA, CABO, CAL, CGM, CPA, EVT, OAX, PET, SMOC, SMOR, SMS, SOC, SON, TAM, YUC. [MTZ].

**Boeberastrum* (A. Gray) Rydb., 1916. 2/2. BCA, CABO, SON. [NEA].

**Boeberoides* (DC.) Strother, 1986. 1/1. BAL, CPA, EVT, SMS. [NEA, MTZ].

**Bolanosa* A. Gray, 1852. 1/1. ALTS, CPA, EVT, SMOC, SMS. [NEA, MTZ].

*Borrichia* Adans., 1763. 3/2. ALTN, ALTS, OAX, TAM, YUC. [MTZ].

*Brickellia* Elliot, 1824. 100/96. ALCH, ALTN, ALTS, BAL, BCA, CABO, CAL, CGM, CPA, EVT, OAX, PET, SMOC, SMOR, SMS, SOC, SON, TAM. [MTZ].

**Calanticaria* (B.L. Rob. & Greenm.) E.E. Schill. & Panero, 2002. 5/5. ALTN, ALTS, EVT, OAX, SMOC, SMOR. [NEA, MTZ].

*Calycoseris* A. Gray, 1853. 2/2. CAL, SON. [NEA].

*Calyptocarpus* Less., 1832. 3/2. ALCH, ALTN, ALTS, BAL, BCA, CGM, CPA, EVT, OAX, PET, SMOC, SMOR, SMS, SOC, TAM, YUC. [MTZ].

*Carminatia* Moç. ex DC., 1838. 4/4. ALCH, ALTN, ALTS, BAL, BCA, CABO, CPA, EVT, OAX, SMOC, SMOR, SMS, SOC, SON. [NEA, MTZ].

*Carphochaete* A. Gray, 1849. 7/7. ALTN, ALTS, EVT, SMOC, SMOR, SMS, SON. [NEA, MTZ].

*Centromadia* Greene, 1894. 4/3. CAL. [NEA].

*Chaenactis* DC., 1836. 18/9. BCA, CAL, SON. [NEA].

*Chaetopappa* DC., 1836. 10/6. ALTN, ALTS, EVT, SMOC, SMOR, SON, TAM. [NEA, MTZ].

**Chaetymenia* Hook. & Arn., 1838. 1/1. ALTS, CPA. [NEA, MTZ].

**Chihuahuana* Urbatsch & R.P. Roberts, 2004. 1/1. ALTN, ALTS. [NEA].

*Chionolaena* DC., 1836. 16/9. ALCH, ALTN, ALTS, EVT, OAX, SMOC, SMOR, SMS, SOC. [NEO, MTZ].

*Chloracantha* G.L. Nesom, Y.B. Suh, D.R. Morgan & B.B. Simpson, 1991. 4/4. ALTN, ALTS, BCA, CABO, CAL, CGM, CPA, EVT, SMOC, SMOR, SMS, SON, TAM. [NEA, MTZ].

**Chromolepis* Benth., 1840. 1/1. ALTN, ALTS, EVT, SMOC. [NEA, MTZ].

*Chrysactinia* A. Gray, 1849. 6/6. ALTN, ALTS, BAL, CGM, EVT, OAX, SMOC, SMOR, TAM. [NEA, MTZ].

*Chrysanthellum* Pers., 1807. 11/10. ALCH, ALTN, ALTS, BAL, CGM, CPA, EVT, OAX, SMOC, SMOR, SMS, SOC. [MTZ].

*Cirsium* L., 1753. 60/45. ALCH, ALTN, ALTS, BAL, BCA, CABO, CAL, CGM, CPA, EVT, OAX, PET, SMOC, SMOR, SMS, SOC, SON, TAM, YUC. [MTZ].

*Clappia* A. Gray, 1859. 1/1. TAM. [NEA].

*Comaclinium* Scheidw. & Planch., 1852. 1/1. CGM, CPA, SOC. [NEO].

*Conoclinium* DC., 1836. 4/3. ALCH, ALTN, ALTS, CGM, CPA, OAX, PET, SMOC, SMOR, SON, TAM, YUC. [NEA, MTZ].

*Coreocarpus* Benth., 1844. 11/11. ALTN, BAL, BCA, CABO, CPA, EVT, SMOC, SON. [NEA, MTZ].

*Coreopsis* L., 1753. 35/21. ALTN, ALTS, BCA, CAL, EVT, OAX, SMOC, SMOR, SMS, TAM. [MTZ].

*Corethrogyne* DC., 1836. 1/1. CAL. [NEA].

*Cosmos* Cav., 1791. 37/37. ALCH, ALTN, ALTS, BAL, BCA, CABO, CGM, CPA, EVT, OAX, PET, SMOC, SMOR, SMS, SOC, SON, YUC. [MTZ].

**Coulterella* Vasey & Rose, 1890. 1/1. BCA, CABO. [NEA].

*Critoniadelphus* R.M. King & H. Rob., 1971. 2/1. ALCH, CGM, CPA, OAX, PET, SMOC, SMOR, SMS, SOC. [NEO, MTZ].

*Cymophora* B.L. Rob., 1907. 5/4. BAL, CPA. [NEO, MTZ].

*Dahlia* Cav., 1791. 43/43. ALCH, ALTN, ALTS, BAL, CGM, CPA, EVT, OAX, SMOC, SMOR, SMS, SOC. [MTZ].

**Damnxanthodium* Strother, 1987. 1/1. SMOC. [NEA, MTZ].

**Davilanthus* E.E. Schill. & Panero, 2010. 7/7. ALTS, EVT, OAX, SMOR. [NEA, MTZ].

*Decachaeta* DC., 1836. 8/8. ALCH, ALTS, BAL, CGM, CPA, EVT, OAX, SMOC, SMOR, SMS, SOC. [NEA, MTZ].

*Deinandra* Greene, 1897. 17/11. BCA, CAL. [NEA].

*Delilia* Spreng., 1823. 2/1. ALCH, ALTS, BAL, CGM, CPA, EVT, OAX, PET, SMOC, SMOR, SMS, SOC, YUC. [NEO, MTZ].

**Dendroviguiera* E.E. Schill. & Panero, 2011. 14/14. ALCH, ALTN, ALTS, BAL, CPA, EVT, OAX, SMOC, SMOR, SMS, SOC. [NEA, MTZ].

*Desmanthodium* Benth., 1872. 8/5. ALCH, BAL, CPA, EVT, OAX, SMOC, SMS, SOC. [NEO, MTZ].

*Dichaetophora* A. Gray, 1849. 1/1. ALTN, ALTS, TAM. [NEA].

*Dicoria* Torr. & A. Gray, 1859. 3/2. CAL, SON. [NEA, MTZ].

*Dicranocarpus* A. Gray, 1855. 1/1. ALTN, ALTS. [NEA, MTZ].

*Dieteria* Nutt., 1840. 5/5. ALTN, ALTS, CAL, SON. [NEA].

**Digitacalia* Pippen, 1968. 6/6. ALCH, ALTS, BAL, CPA, EVT, OAX, SMOC, SMOR, SMS, SOC. [NEA, MTZ].

**Dugesia* A. Gray, 1882. 1/1. ALTS, EVT, OAX, SMOR. [NEA, MTZ].

**Dyscritothamnus* B.L. Rob., 1922. 2/2. ALTS. [NEA, MTZ].

*Dyssodia* Cav., 1802. 6/5. ALCH, ALTN, ALTS, BAL, CGM, CPA, EVT, OAX, SMOC, SMOR, SMS, SOC, SON, TAM. [NEA, MTZ].

*Electranthera* Mesfin, D.J. Crawford & Pruski, 2015. 12/12. ALCH, ALTS, BAL, CGM, CPA, EVT, OAX, PET, SMOC, SMOR, SMS, SOC. [NEA, MTZ].

*Encelia* Adans., 1763. 21/13. ALTN, ALTS, BCA, CABO, CAL, CPA, SON. [NEA, MTZ].

*Engelmannia* Torr. & A. Gray ex Nutt., 1840. 1/1. ALTN, ALTS, TAM. [NEA, MTZ].

*Eremosis* (DC.) Gleason, 1906. 27/21. ALCH, ALTN, ALTS, BAL, CGM, CPA, EVT, OAX, PET, SMOC, SMOR, SMS, SOC, SON, TAM, YUC. [NEA, MTZ].

**Eryngiophyllum* Greenm., 1903. 2/2. BAL, CPA. [NEA, MTZ].

*Espejoa* DC., 1836. 1/1. CGM, CPA, SOC. [NEO].

*Eupatoriastrum* Greenm., 1903. 7/7. ALCH, BAL, CGM, CPA, EVT, OAX, SMOC, SMS, SOC. [NEA, MTZ].

**Euphrosyne* DC., 1836. 1/1. ALTN, ALTS. [NEA, MTZ].

**Eutetras* A. Gray, 1879. 2/2. ALTS, EVT, SMOC, SMOR. [NEA, MTZ].

**Faxonia* Brandegee, 1894. 1/1. CABO. [NEA].

*Flaveria* Juss., 1789. 24/16. ALCH, ALTN, ALTS, BAL, BCA, CGM, CPA, EVT, OAX, PET, SMOC, SMOR, SMS, SOC, SON, TAM, YUC. [MTZ].

*Florestina* Cass., 1817. 8/8. ALCH, ALTN, ALTS, BAL, CGM, CPA, EVT, OAX, SMOC, SMOR, SMS, SOC, TAM. [NEA, MTZ].

*Flyriella* R.M. King & H. Rob., 1972. 4/4. ALTN, ALTS, CGM, SMOC, SMOR. [NEA, MTZ].

*Gaillardia* Foug., 1786. 21/14. ALTN, ALTS, EVT, OAX, SMOC, SMOR, SON, TAM. [MTZ].

*Galeana* Llave, 1824. 1/1. ALCH, ALTN, ALTS, BAL, CGM, CPA, EVT, OAX, SMOC, SMS, SOC. [NEO, MTZ].

*Galinsoga* Ruiz & Pav., 1794. 12/8. ALCH, ALTN, ALTS, BAL, BCA, CABO, CGM, CPA, EVT, OAX, PET, SMOC, SMOR, SMS, SOC, SON, YUC. [MTZ].

**Geissolepis* B.L. Rob., 1892. 1/1. ALTS. [NEA, MTZ].

*Geraea* Torr. & A. Gray, 1847. 2/2. BCA, CAL, SON. [NEA].

*Goldmanella* Greenm., 1908. 1/1. CGM, PET, YUC. [NEO].

*Gonzalezia* E.E. Schill. & Panero, 2011. 3/3. ALTN, ALTS, SMOC. [MTZ].

**Greenmaniella* W.M. Sharp, 1935. 1/1. ALTN, ALTS, SMOR, TAM. [NEA, MTZ].

*Grindelia* Willd., 1807. 41/31. ALTN, ALTS, CAL, CGM, EVT, OAX, SMOC, SMOR, SMS, SON. [MTZ].

*Guardiola* Cerv. ex Humb. & Bonpl., 1807. 14/12. ALTN, ALTS, BAL, CPA, EVT, SMOC, SMS, SON. [NEA, MTZ].

*Gundlachia* A. Gray, 1880. 3/3. ALTN, ALTS, BCA, CABO, CPA, SON. [NEA, MTZ].

**Gymnolaena* (DC.) Rydb., 1915. 3/3. BAL, CPA, OAX, SOC. [NEA, MTZ].

*Gymnosperma* Less., 1832. 1/1. ALCH, ALTN, ALTS, BAL, CGM, CPA, EVT, OAX, SMOC, SMOR, SMS, SOC, SON, TAM. [MTZ].

*Haploesthes* A. Gray, 1849. 4/4. ALTN, ALTS. [NEA].

*Harleya* S.F. Blake, 1932. 1/1. CGM, PET, YUC. [NEO].

*Hazardia* Greene, 1887. 11/9. BCA, CABO, CAL. [NEA].

*Hedosyne* Strother, 2000. 1/1. ALTN, ALTS, SON. [NEA, MTZ].

*Helianthella* Torr. & A. Gray, 1842. 6/6. ALTN, ALTS, OAX, SMOC, SMOR, SON. [NEA, MTZ].

*Heliomeris* Nutt., 1848. 5/5. ALCH, ALTN, ALTS, BAL, CPA, EVT, OAX, SMOC, SMOR, SMS, SON. [NEA, MTZ].

*Heliopsis* Pers., 1807. 18/12. ALCH, ALTN, ALTS, BAL, BCA, CABO, CGM, CPA, EVT, OAX, SMOC, SMOR, SMS, SOC, SON, TAM. [MTZ].

**Henricksonia* B.L. Turner, 1977. 1/1. ALTN, ALTS. [NEA].

*Hidalgoa* Llave, 1824. 4/3. ALCH, CGM, CPA, OAX, SMOR, SOC. [NEO].

**Hofmeisteria* Walp., 1846. 12/12. ALCH, ALTS, BAL, BCA, CABO, CGM, CPA, EVT, OAX, SMOC, SMS, SOC, SON. [NEA, MTZ].

**Hybridella* Cass., 1821. 2/2. ALTN, ALTS, EVT, SMOC, SMOR. [NEA, MTZ].

**Hydropectis* Rydb., 1916. 3/3. ALTN, ALTS, SMOC. [NEA, MTZ].

*Hymenopappus* L'Her., 1788. 13/7. ALTN, ALTS, CAL, SMOC, SMOR, SON, TAM. [NEA, MTZ].

*Hymenostephium* Benth., 1873. 11/11. ALCH, ALTS, BAL, CGM, CPA, EVT, OAX, SMOC, SMOR, SMS, SOC. [NEO, MTZ].

*Hymenothrix* A. Gray, 1849. 6/5. ALTN, ALTS, CAL, SMOC, SON. [NEA, MTZ].

**Iostephane* Benth., 1873. 3/3. ALCH, ALTN, ALTS, BAL, CPA, EVT, OAX, SMOC, SMOR, SMS, SOC. [NEA, MTZ].

*Isocoma* Nutt., 1840. 16/16. ALTN, ALTS, BCA, CAL, EVT, OAX, SMOC, SMOR, SON, TAM. [NEA, MTZ].

*Jaegeria* Kunth, 1820. 11/8. ALCH, ALTN, ALTS, BAL, CGM, CPA, EVT, OAX, SMOC, SMOR, SMS, SOC. [NEO, MTZ].

**Jaliscoa* S. Watson, 1890. 3/3. ALTS, BAL, CPA, EVT, SMOC, SMS. [NEA, MTZ].

*Jaumea* Pers., 1807. 2/1. CAL. [NEA].

*Jefea* Strother, 1991. 4/4. ALTN, ALTS, EVT, OAX, SMOR, TAM. [NEA, MTZ].

*Kyrsteniopsis* R.M. King & H. Rob., 1971. 9/9. ALCH, ALTS, BAL, CGM, CPA, EVT, OAX, SMOR, SMS, SOC. [NEO, MTZ].

*Laennecia* Cass., 1822. 18/12. ALCH, ALTN, ALTS, BAL, BCA, CABO, CAL, CGM, CPA, EVT, OAX, SMOC, SMOR, SMS, SOC, SON, TAM. [MTZ].

*Lagascea* Cav., 1803. 9/9. ALCH, ALTN, ALTS, BAL, CGM, CPA, EVT, OAX, PET, SMOC, SMOR, SMS, SOC, SON, YUC. [MTZ].

*Lasianthaea* DC., 1836. 15/25. ALCH, ALTN, ALTS, BAL, CGM, CPA, EVT, OAX, PET, SMOC, SMOR, SMS, SOC, SON, TAM, YUC. [MTZ].

**Leiboldia* Schltdl. ex Gleason, 1906. 2/2. EVT, OAX, SMOR. [NEA, MTZ].

*Lepidonia* S.F. Blake, 1936. 9/6. BAL, CGM, CPA, OAX, SMOR, SMS, SOC. [NEO, MTZ].

**Leucactinia* Rydb., 1915. 1/1. ALTN, ALTS. [NEA, MTZ].

*Leuciva* Rydb., 1922. 1/1. ALTN, ALTS, SON. [NEA, MTZ].

*Leucosyris* Greene, 1897. 9/8. ALTN, ALTS, BCA, SON. [NEA, MTZ].

**Liabellum* Rydb., 1927. 5/5. ALTN, ALTS, EVT, SMOC, SMS. [NEA, MTZ].

*Lindheimera* A. Gray & Engelm., 1846. 1/1. ALTN. [NEA].

**Loxothysanus* B.L. Rob., 1907. 2/2. ALCH, ALTS, CGM, CPA, EVT, OAX, SMOR, SMS, SOC. [NEA, MTZ].

*Lundellianthus* H. Rob., 1978. 8/4. ALCH, CGM, EVT, PET, SOC, YUC. [NEO, MTZ].

*Machaeranthera* Nees, 1832. 2/2. ALTN, ALTS, CPA, SON. [NEA, MTZ].

*Macvaughiella* R.M. King & H. Rob., 1968. 2/2. ALCH, CGM, CPA, OAX, SOC. [NEO].

*Malacothrix* DC., 1838. 19/10. ALTN, BCA, CABO, CAL, SMOC, SON. [NEA].

*Malperia* S. Watson, 1889. 1/1. BCA, CAL, SON. [NEA].

**Marshalljohnstonia* Henr., 1976. 1/1. ALTN. [NEA].

**Medranoa* Urbatsch & R.P. Roberts, 2004. 1/1. ALTN, ALTS, SMOR. [NEA].

*Megaliabum* Rydb., 1927. 2/2. ALCH, ALTS, CGM, CPA, EVT, OAX, SMOC, SMS, SOC. [NEA, MTZ].

*Melampodium* L., 1753. 43/43. ALCH, ALTN, ALTS, BAL, BCA, CABO, CGM, CPA, EVT, OAX, PET, SMOC, SMOR, SMS, SOC, SON, TAM, YUC. [MTZ].

**Mexerion* G.L. Nesom, 1990. 2/2. EVT, OAX, SMOC. [NEA, MTZ].

**Mexianthus* B.L. Rob., 1928. 1/1. CPA. [NEA, MTZ].

**Microspermum* Lag., 1816. 9/9. EVT, OAX, SMS, SOC. [NEA, MTZ].

*Milleria* L., 1753. 2/2. ALCH, ALTN, ALTS, BAL, CABO, CGM, CPA, EVT, OAX, PET, SMOC, SMOR, SMS, SOC, SON, YUC. [NEO, MTZ].

*Monoptilon* Torr. & A. Gray, 1844. 2/1. CAL, SON. [NEA].

*Montanoa* Cerv., 1825. 35/23. ALCH, ALTN, ALTS, BAL, CGM, CPA, EVT, OAX, PET, SMOC, SMOR, SMS, SOC, SON, YUC. [NEO, MTZ].

*Nahuatlea* V.A. Funk, 2017. 7/7. ALTN, ALTS, BAL, BCA, CABO, CGM, EVT, OAX, SMOR, SMS, SON, TAM. [NEA, MTZ].

*Nelsonianthus* H. Rob. & Brettell, 1973. 2/2. EVT, OAX, SMOR, SOC. [NEO].

*Neonesomia* Urbatsch & R.P. Roberts, 2004. 2/2. ALTS, SMOR, TAM. [NEA, MTZ].

*Neurolaena* R. Br., 1817. 12/11. ALCH, BAL, CGM, CPA, OAX, PET, SMOR, SMS, SOC, TAM, YUC. [NEO, MTZ].

*Nicolletia* A. Gray, 1845. 3/3. ALTN, ALTS, BCA, SON. [NEA, MTZ].

*Oreochrysum* Rydb., 1906. 1/1. SMOC, SON. [NEA, MTZ].

*Orthopappus* Gleason, 1906. 1/1. ALCH, CGM, CPA, PET, SMOR, SMS, SOC. [NEO, MTZ].

*Osbertia* Greene, 1895. 4/2. ALCH, ALTN, ALTS, EVT, OAX, SMOR, SMS, SOC, TAM. [NEO, MTZ].

*Oteiza* Llave, 1832. 4/3. ALTS, EVT, OAX, SMOR, SMS. [NEA, MTZ].

*Otopappus* Benth., 1873. 17/14. ALCH, ALTS, BAL, CGM, CPA, EVT, OAX, PET, SMOC, SMOR, SMS, SOC, YUC. [NEO, MTZ].

*Oxylobus* (Moç. ex DC.) A. Gray, 1879. 7/7. ALCH, EVT, OAX, SMOR, SMS, SOC. [NEO, MTZ].

**Oxypappus* Benth., 1845. 1/1. ALTN, ALTS, EVT, OAX, SMOC, SMS. [NEA, MTZ].

*Palafoxia* Lag., 1816. 11/10. ALTN, ALTS, BCA, CABO, CAL, CGM, CPA, OAX, SON, TAM. [NEA, MTZ].

**Paneroa* E.E. Schill., 2008. 1/1. OAX, SMS. [NEA, MTZ].

*Parthenice* A. Gray, 1853. 2/2. ALTN, BCA, CPA, SON. [NEA, MTZ].

*Parthenium* L., 1753. 16/12. ALCH, ALTN, ALTS, BAL, CABO, CGM, CPA, EVT, OAX, PET, SMOC, SMOR, SMS, SOC, SON, TAM, YUC. [MTZ].

*Pectis* L., 1759. 92/56. ALCH, ALTN, ALTS, BAL, BCA, CABO, CAL, CGM, CPA, EVT, OAX, PET, SMOC, SMOR, SMS, SOC, SON, TAM, YUC. [MTZ].

**Pelucha* S. Watson, 1889. 1/1. BCA, SON. [NEA].

*Pericome* A. Gray, 1853. 3/2. ALTN, EVT, SMOC, SON. [NEA, MTZ].

*Perityle* Benth., 1844. 66/47. ALCH, ALTN, ALTS, BCA, CABO, CAL, CGM, CPA, EVT, SMOC, SMOR, SMS, SOC, SON, TAM. [MTZ].

*Perymenium* Schrad., 1830. 62/56. ALCH, ALTN, ALTS, BAL, CGM, CPA, EVT, OAX, PET, SMOC, SMOR, SMS, SOC, YUC. [NEO, MTZ].

*Peteravenia* R.M. King & H. Rob., 1971. 5/3. ALCH, ALTN, ALTS, BAL, CGM, CPA, EVT, OAX, PET, SMOR, SMS, SOC. [NEO, MTZ].

*Peucephyllum* A. Gray, 1859. 1/1. BCA, CAL, SON. [NEA].

*Philactis* Schrad., 1833. 2/2. ALCH, BAL, CGM, CPA, OAX, SOC. [NEO, MTZ].

*Pinaropappus* Less., 1832. 13/13. ALCH, ALTN, ALTS, BAL, CGM, CPA, EVT, OAX, SMOC, SMOR, SMS, SOC, SON, TAM. [NEA, MTZ].

**Pippenalia* McVaugh, 1972. 1/1. ALTN, ALTS, EVT, SMOC. [NEA, MTZ].

*Piqueria* Cav., 1794. 6/6. ALCH, ALTN, ALTS, BAL, CGM, CPA, EVT, OAX, SMOC, SMOR, SMS, SOC. [NEO, MTZ].

*Pittocaulon* H. Rob. & Brettell, 1973. 7/6. ALCH, ALTS, BAL, CGM, CPA, EVT, OAX, SMOC, SMOR, SMS, SOC. [NEA, MTZ].

**Plagiolophus* Greenm., 1904. 1/1. PET, YUC. [NEA].

*Plateilema* (A. Gray) Cockerell, 1904. 1/1. ALTN, ALTS. [NEA].

*Pleurocoronis* R.M. King & H. Rob., 1966. 3/3. BCA, CAL, SON. [NEA, MTZ].

*Podachaenium* Benth., 1852. 6/4. ALCH, ALTS, BAL, CGM, CPA, EVT, OAX, PET, SMOC, SMOR, SMS, SOC. [NEA, MTZ].

*Porophyllum* Adans., 1763. 25/17. ALCH, ALTN, ALTS, BAL, BCA, CABO, CAL, CGM, CPA, EVT, OAX, PET, SMOC, SMOR, SMS, SOC, SON, YUC. [MTZ].

*Prenanthella* Rydb., 1906. 1/1. CAL, SON. [NEA].

**Psacaliopsis* H. Rob. & Brettell, 1974. 5/5. OAX, SMS. [NEA, MTZ].

*Psacalium* Cass., 1826. 51/50. ALCH, ALTN, ALTS, BAL, CPA, EVT, OAX, SMOC, SMOR, SMS, SOC, SON. [NEA, MTZ].

*Psathyrotopsis* Rydb., 1927. 3/3. ALTN, ALTS. [NEA].

*Pseudelephantopus* Rohr, 1792. 2/1. ALCH, ALTS, BAL, CGM, CPA, EVT, OAX, PET, SMOC, SMOR, SMS, SOC, YUC. [MTZ].

*Pseudoclappia* Rydb., 1923. 2/1. ALTN. [NEA].

*Psilactis* A. Gray, 1849. 6/5. ALCH, ALTN, ALTS, BAL, CGM, EVT, OAX, SMOC, SMOR, SMS, SON, TAM. [MTZ].

*Psilostrophe* DC., 1838. 7/5. ALTN, ALTS, BCA, SMOC, SMOR, SON. [NEA, MTZ].

*Rafinesquia* Nutt., 1841. 2/2. ALTN, BCA, CAL, SON. [NEA].

*Ratibida* Raf., 1817. 7/6. ALTN, ALTS, SMOC, SMOR, SON, TAM. [NEA, MTZ].

*Rensonia* S.F. Blake, 1923. 1/1. CGM, CPA, SOC. [NEO].

*Robinsonecio* T.M. Barkley & J.P. Janovec, 1996. 2/1. EVT, OAX, SMOR, SOC. [NEO, MTZ].

*Rojasianthe* Standl. & Steyerm., 1940. 1/1. ALCH, SOC. [NEO].

*Roldana* Llave, 1825. 57/57. ALCH, ALTN, ALTS, BAL, CGM, CPA, EVT, OAX, SMOC, SMOR, SMS, SOC, SON, TAM. [NEA, MTZ].

*Rumfordia* DC., 1836. 10/10. ALCH, ALTN, ALTS, BAL, CABO, CGM, CPA, EVT, OAX, SMOC, SMOR, SMS, SOC, TAM. [NEO, MTZ].

*Sabazia* Cass., 1827. 11/11. ALCH, ALTN, ALTS, BAL, CABO, CPA, EVT, OAX, SMOR, SMS, SOC. [NEA, MTZ].

**Sanrobertia* Nesom, 2018. 1/1. ALTS. [NEA].

*Sanvitalia* Lam., 1792. 7/5. ALCH, ALTN, ALTS, BAL, CGM, CPA, EVT, OAX, PET, SMOC, SMOR, SMS, SOC, SON, TAM, YUC. [MTZ].

*Sartwellia* A. Gray, 1852. 4/3. ALTN, ALTS. [NEA, MTZ].

*Schistocarpha* Less., 1831. 12/8. ALCH, BAL, CGM, CPA, EVT, OAX, PET, SMOR, SMS, SOC. [NEO, MTZ].

*Schkuhria* Roth, 1797. 5/3. ALCH, ALTN, ALTS, BAL, CGM, CPA, EVT, OAX, SMOC, SMOR, SMS, SOC, SON. [MTZ].

*Sclerocarpus* Jacq., 1787. 9/6. ALCH, ALTN, ALTS, BAL, BCA, CABO, CGM, CPA, EVT, OAX, PET, SMOC, SMOR, SMS, SOC, TAM, YUC. [MTZ].

**Selloa* Kunth, 1820. 1/1. EVT, OAX. [NEA, MTZ].

*Shinnersia* R.M. King & H. Rob., 1970. 1/1. ALTN. [NEA].

*Sidneya* E.E. Schill. & Panero, 2011. 3/3. ALTN, ALTS, BAL, CGM, CPA, OAX, SON, TAM. [MTZ].

*Simsia* Pers., 1807. 20/20. ALCH, ALTN, ALTS, BAL, CGM, CPA, EVT, OAX, PET, SMOC, SMOR, SMS, SOC, TAM, YUC. [MTZ].

*Sinclairia* Hook. & Arn., 1841. 29/17. ALCH, ALTN, ALTS, BAL, CGM, CPA, EVT, OAX, PET, SMOC, SMOR, SMS, SOC. [NEO, MTZ].

*Sinclairiopsis* Rydb., 1927. 2/2. BAL, CGM, CPA, OAX, SMOR, SMS. [NEO, MTZ].

**Sphaeromeria* Nutt., 1841. 1/1. BCA. [NEA].

*Spiracantha* Kunth, 1820. 1/1. OAX, YUC. [NEO, MTZ].

*Squamopappus* R.K. Jansen, N.A. Harriman & Urbatsch, 1982. 1/1. ALCH, SOC. [NEO].

*Stebbinsoseris* K.L. Chambers, 1991. 2/1. CAL. [NEA].

**Stenocarpha* S.F. Blake, 1915. 2/2. CPA, SMOC. [NEA, MTZ].

**Stephanodoria* Greene, 1895. 1/1. ALTS. [NEA, MTZ].

*Stephanomeria* Nutt., 1841. 17/10. ALTN, BCA, CAL, SMOC, SON. [NEA, MTZ].

*Stevia* Cav., 1797. 240/129. ALCH, ALTN, ALTS, BAL, BCA, CABO, CGM, CPA, EVT, OAX, PET, SMOC, SMOR, SMS, SOC, SON, TAM. [MTZ].

*Steviopsis* R.M. King & H. Rob., 1971. 7/7. ALTN, ALTS, BAL, CPA, EVT, OAX, SMOC, SMOR, SMS, SON. [NEA, MTZ].

**Stramentopappus* H. Rob. & V.A. Funk, 1987. 2/2. OAX. [NEA].

**Strotheria* B.L. Turner, 1972. 1/1. ALTN, ALTS. [NEA].

*Struchium* P. Browne, 1756. 1/1. CGM, PET, SOC. [NEO].

*Synedrella* Gaertn., 1791. 1/1. ALCH, BAL, CGM, CPA, OAX, PET, SMOC, SMOR, SMS, SOC, YUC. [MTZ].

*Tagetes* L., 1753. 45/28. ALCH, ALTN, ALTS, BAL, BCA, CABO, CGM, CPA, EVT, OAX, PET, SMOC, SMOR, SMS, SOC, SON, TAM, YUC. [MTZ].

*Tamaulipa* R.M. King & H. Rob., 1971. 1/1. ALTN, ALTS, TAM. [NEA, MTZ].

**Tehuana* Panero & Villaseñor, 1996. 1/1. CPA. [NEA].

*Telanthophora* H. Rob. & Brettell, 1974. 9/7. ALCH, BAL, CGM, CPA, EVT, OAX, PET, SMOR, SMS, SOC. [NEO, MTZ].

*Tetrachyron* Schltr., 1847. 9/9. ALCH, ALTS, EVT, OAX, SMOR, SOC. [NEA, MTZ].

*Thelesperma* Less., 1831. 14/12. ALTN, ALTS, SMOC, SMOR, SON, TAM. [MTZ].

*Thymophylla* Lag., 1816. 15/15. ALTN, ALTS, BAL, BCA, CAL, CGM, CPA, EVT, OAX, SMOC, SMOR, SON, TAM. [NEA, MTZ].

*Tithonia* Desf., 1789. 13/12. ALCH, ALTN, ALTS, BAL, CGM, CPA, EVT, OAX, PET, SMOC, SMOR, SMS, SOC, SON, TAM, YUC. [MTZ].

**Tomentaurum* G.L. Nesom, 1991. 1/1. SMOC. [NEA, MTZ].

*Trichocoronis* A. Gray, 1849. 3/3. BAL, BCA, OAX, TAM. [NEA, MTZ].

**Trichocoryne* S.F. Blake, 1924. 1/1. SMOC. [NEA, MTZ].

*Trichoptilium* A. Gray, 1859. 1/1. BCA, CAL, SON. [NEA].

*Trichospira* Kunth, 1820. 1/1. OAX, SOC. [NEO].

*Tridax* L., 1753. 31/27. ALCH, ALTN, ALTS, BAL, CGM, CPA, EVT, OAX, PET, SMOC, SMOR, SMS, SOC, TAM, YUC. [MTZ].

*Trigonospermum* Less., 1832. 6/6. ALCH, ALTN, ALTS, BAL, CGM, CPA, EVT, OAX, SMOC, SMOR, SMS, SOC. [NEA, MTZ].

*Tuxtla* Villaseñor & Strother, 1989. 1/1. CGM. [NEO].

**Urbinella* Greenm., 1903. 1/1. SMOC. [NEA, MTZ].

*Uropappus* Nutt., 1841. 1/1. ALTN, BCA, CAL, SON. [NEA].

*Varilla* A. Gray, 1849. 3/3. ALTN, ALTS, TAM. [NEA].

*Venegasia* DC., 1838. 1/1. CAL. [NEA].

*Verbesina* L., 1753. 250/170. ALCH, ALTN, ALTS, BAL, BCA, CABO, CAL, CGM, CPA, EVT, OAX, PET, SMOC, SMOR, SMS, SOC, SON, TAM, YUC. [MTZ].

*Vernonia* Schreb., 1791. 20/16. ALTN, ALTS, BAL, CGM, CPA, EVT, OAX, PET, SMOC, SMOR, SMS, TAM. [MTZ].

**Vigethia* W.A. Weber, 1943. 1/1. SMOR. [NEA, MTZ].

**Villasenoria* B.L. Clark, 1999. 1/1. CGM, OAX, SOC. [NEA, MTZ].

*Wamalchitamia* Strother, 1991. 5/4. ALCH, BAL, CGM, CPA, SMOC, SMS, SOC. [NEO, MTZ].

*Xanthisma* DC., 1836. 17/17. ALTN, ALTS, BCA, CABO, CAL, CGM, CPA, EVT, OAX, SMOC, SMOR, SON, TAM. [NEA, MTZ].

*Xanthium* L., 1753. 3/3. ALCH, ALTN, ALTS, BAL, BCA, CABO, CAL, CGM, CPA, EVT, OAX, SMOC, SMOR, SMS, SOC, SON, TAM. [MTZ].

*Xanthocephalum* Willd., 1807. 6/6. ALTN, ALTS, EVT, OAX, SMOC, SMOR, SON. [NEA, MTZ].

**Xylovirgata* Urbatsch & R.P. Roberts, 2004. 1/1. ALTN, ALTS. [NEA].

*Zaluzania* Pers., 1807. 13/13. ALTN, ALTS, BAL, CPA, EVT, OAX, SMOC, SMOR, SMS, SON. [NEA, MTZ].

**Zandera* D.L. Schulz, 1988. 3/3. BAL, EVT, SMOC, SMS. [NEA, MTZ].

*Zinnia* L., 1759. 26/26. ALCH, ALTN, ALTS, BAL, CGM, CPA, EVT, OAX, PET, SMOC, SMOR, SMS, SOC, SON, TAM, YUC. [MTZ].

*Zyzyxia* Strother, 1991. 1/1. ALCH, CGM, PET. [NEO].

**Balanophoraceae**

*Helosis* Rich., 1822. 3/2. ALCH, BAL, CGM, CPA, OAX, PET, SMOR, SMS, SOC. [NEO, MTZ].

**Bataceae**

*Batis* L., 1759. 2/1. BCA, CABO, CAL, CGM, CPA, OAX, PET, SOC, SON, TAM, YUC. [MTZ].

**Bignoniaceae**

*Amphitecna* Miers, 1868. 20/11. ALCH, BAL, CGM, CPA, EVT, OAX, PET, SMOR, SOC, YUC. [MTZ].

*Astianthus* Don, 1823. 1/1. ALTS, BAL, CGM, CPA, OAX, PET, SOC. [NEO, MTZ].

*Callichlamys* Miq., 1845. 1/1. CGM, CPA, PET, SOC. [NEO].

*Chilopsis* Don, 1823. 3/3. ALTN, ALTS, BCA, CAL, SMOC, SMOR, SON, TAM. [NEA, MTZ].

*Crescentia* L., 1753. 2/2. ALCH, ALTN, ALTS, BAL, BCA, CABO, CGM, CPA, EVT, OAX, PET, SMOC, SMOR, SMS, SOC, SON, YUC. [MTZ].

*Godmania* Hemsl., 1879. 2/1. ALCH, BAL, CGM, CPA, EVT, OAX, PET, SMOC, SMS, SOC, YUC. [NEO, MTZ].

*Roseodendron* Miranda, 1965. 2/1. BAL, CGM, CPA, OAX, PET, SOC. [NEO, MTZ].

*Stizophyllum* Miers, 1863. 3/2. CGM, OAX, PET, SOC, YUC. [NEO].

*Tourrettia* Foug., 1787. 1/1. CPA, SOC. [NEO].

**Bixaceae**

*Amoreuxia* Moç. & Sessé ex DC., 1825. 4/4. ALCH, ALTN, ALTS, BAL, BCA, CABO, CGM, CPA, EVT, OAX, PET, SMOC, SMOR, SMS, SOC, SON, TAM, YUC. [MTZ].

**Boraginaceae**

*Antiphytum* DC. ex Meisn., 1840. 12/9. ALTN, ALTS, BAL, BCA, CPA, EVT, OAX, SMOC, SMOR, SMS. [MTZ].

*Harpagonella* A. Gray, 1876. 2/1. BCA, CAL, SON. [NEA].

*Johnstonella* Brand, 1925. 12/9. ALTN, BCA, CABO, CAL, CPA, SON. [MTZ].

**Lasiarrhenum* I.M. Johnst., 1924. 1/1. SMOC. [NEA, MTZ].

*Lithospermum* L., 1753. 60/46. ALCH, ALTN, ALTS, BAL, CGM, CPA, EVT, OAX, SMOC, SMOR, SMS, SOC, SON, TAM. [MTZ].

**Mimophytum* Greenm., 1905. 3/3. CGM, SMOR. [NEA, MTZ].

**Oncaglossum* Sutorý, 2010. 1/1. BAL, CPA, EVT, SMS. [NEA, MTZ].

*Pectocarya* DC. ex Meisn., 1840. 10/7. ALTN, BCA, CAL, SON. [NEA].

**Brassicaceae**

**Asta* Klotzsch ex O.E. Schulz, 1933. 3/3. ALTN, ALTS, SMOR. [NEA, MTZ].

*Athysanus* Greene, 1885. 2/2. BCA, CAL. [NEA].

*Cakile* Mill., 1754. 7/5. BCA, CAL, CGM, PET, TAM, YUC. [MTZ].

**Chaunanthus* O.E. Schulz, 1924. 4/4. CPA, OAX. [NEA, MTZ].

**Cibotarium* O.E. Schulz, 1933. 2/2. ALTN, ALTS. [NEA].

*Coelophragmus* O.E. Schulz, 1924. 1/1. ALTN, ALTS, CPA. [NEA, MTZ].

*Dithyrea* Harv., 1845. 3/3. ALTN, BCA, CAL, SON. [NEA].

*Dryopetalon* A. Gray, 1853. 9/8. ALTN, ALTS, BCA, CABO, CPA, SMOC, SMOR, SON. [NEA, MTZ].

*Exhalimolobos* Al-Shehbaz & C.D. Bailey, 2007. 9/5. ALTS, BAL, EVT, SMOR, SMS. [NEO, MTZ].

*Halimolobos* Wittst., 1852. 7/7. ALTN, ALTS, SMOC, SMOR. [MTZ].

**Lexarzanthe* N. Diego & Calderón, 2004. 1/1. SMS. [NEA].

*Lyrocarpa* Hook. & Harv. ex Harv., 1845. 4/4. BCA, CABO, CAL, SON. [NEA].

*Mancoa* Wedd., 1859. 9/5. ALTN, ALTS, EVT, SMOR. [NEO, MTZ].

*Nerisyrenia* Greene, 1900. 10/10. ALTN, ALTS, SMOR. [NEA, MTZ].

**Ornithocarpa* Rose, 1905. 2/2. ALTS, SMOC. [NEA, MTZ].

*Pennellia* Nieuwl., 1918. 10/5. ALTN, ALTS, EVT, OAX, SMOC, SMOR, SOC, SON. [MTZ].

*Phravenia* Al-Shehbaz & S.I. Warwick, 2011. 1/1. ALTN, ALTS. [NEA].

*Planodes* Greene, 1912. 2/2. ALTS, EVT. [NEA, MTZ].

**Rhaphanorhyncha* Rollins, 1976. 1/1. ALTN. [NEA].

*Romanschulzia* O.E. Schulz, 1933. 13/8. ALTS, EVT, OAX, SMOC, SMOR, SMS. [NEA, MTZ].

**Scoliaxon* Payson, 1924. 1/1. ALTN, ALTS. [NEA].

*Sibara* Greene, 1896. 6/6. ALTN, ALTS, BCA, TAM. [NEA].

**Sphaerocardamum* Schauer, 1847. 4/4. ALTN, ALTS, SMOR. [NEA, MTZ].

*Streptanthella* Rydb., 1917. 2/1. CAL, SON. [NEA].

*Synthlipsis* A. Gray, 1849. 2/2. ALTN, ALTS, SMOR, TAM. [NEA, MTZ].

*Thelypodiopsis* Rydb., 1907. 8/4. ALTN, ALTS, CGM, SMOC. [NEA, MTZ].

*Thysanocarpus* Hook., 1830. 4/4. BCA, CAL, SON. [NEA].

*Tomostima* Raf., 1825. 6/3. ALTN, ALTS, BCA, CAL, CPA, SON. [MTZ].

**Bromeliaceae**

*Androlepis* Brongn. ex Houllet, 1870. 2/1. CGM, PET, SOC. [NEO].

*Bakerantha* L.B. Sm., 1934. 4/4. ALTS, BAL, CGM, SMOR [NEA].

*Catopsis* Griseb., 1864. 18/16. ALCH, BAL, CGM, CPA, EVT, OAX, PET, SMOC, SMOR, SMS, SOC, YUC. [MTZ].

*Hechtia* Klotzsch, 1835. 76/76. ALCH, ALTN, ALTS, BAL, BCA, CABO, CGM, CPA, EVT, OAX, PET, SMOC, SMOR, SMS, SOC, SON, TAM, YUC. [NEA, MTZ].

*Hohenbergiopsis* L.B. Sm. & Read, 1976. 1/1. OAX. [NEO].

**Ursulaea* Read & Baensch, 1994. 2/2. CPA, SMOC, SMS. [NEA, MTZ].

*Viridantha* Espejo, 2002. 8/8. ALCH, ALTS, BAL, CGM, CPA, EVT, OAX, SMOC, SMOR, SMS. [NEA, MTZ].

**Burmanniaceae**

*Apteria* Nutt., 1834. 1/1. ALCH, CGM, OAX, SMOR, SOC. [NEO].

*Dictyostega* Miers, 1840. 1/1. ALCH, OAX, SMOR, SOC. [NEO, MTZ].

**Burseraceae**

**Beiselia* Forman, 1987. 1/1. BAL, CPA. [NEA].

*Bursera* Jacq., 1762. 120/101. ALCH, ALTN, ALTS, BAL, BCA, CABO, CAL, CGM, CPA, EVT, OAX, PET, SMOC, SMOR, SMS, SOC, SON, TAM, YUC. [MTZ].

**Cabombaceae**

*Brasenia* Schreb., 1789. 1/1. OAX. [MTZ].

**Cactaceae**

**Acharagma* (N.P. Taylor) Glass, 1997. 2/2. ALTN, ALTS. [NEA].

*Ariocarpus* Scheidw., 1838. 7/6. ALTN, ALTS, SMOR. [NEA, MTZ].

*Astrophytum* Lem., 1839. 6/5. ALTN, ALTS, EVT, SMOR, TAM. [NEA, MTZ].

**Aztekium* Boed., 1929. 2/2. ALTN, ALTS. [NEA, MTZ].

**Backebergia* Bravo, 1953. 1/1. BAL, CPA. [NEA, MTZ].

*Bergerocactus* Britton & Rose, 1909. 1/1. BCA, CAL. [NEA].

*Carnegiea* Britton & Rose, 1908. 1/1. ALTN, CPA, SON. [NEA, MTZ].

**Cephalocereus* Pfeiff., 1838. 15/15. ALCH, ALTS, BAL, CGM, CPA, EVT, OAX, SMOR, SMS, SOC. [NEA, MTZ].

**Cochemiea* Walton, 1899. 5/5. BCA, CABO. [NEA].

*Coryphantha* Lem., 1868. 54/54. ALTN, ALTS, BAL, CGM, CPA, EVT, OAX, SMOC, SMOR, SMS, SON, TAM. [NEA, MTZ].

**Cumarinia* Buxb., 1951. 1/1. ALTN, ALTS. [NEA, MTZ].

*Cylindropuntia* (Engelm.) F.M. Knuth, 1935. 42/42. ALTN, ALTS, BAL, BCA, CABO, CAL, CGM, CPA, EVT, OAX, SMOC, SMOR, SON, TAM. [MTZ].

*Deamia* Britton & Rose, 1920. 4/3. ALCH, CGM, CPA, EVT, OAX, PET, SOC, YUC. [NEO].

*Disocactus* Lindl., 1845. 10/10. ALCH, ALTS, BAL, CGM, CPA, EVT, OAX, SMOC, SMOR, SMS, SOC. [NEA, MTZ].

*Echinocactus* Link & Otto, 1827. 6/6. ALTN, ALTS, CAL, EVT, OAX, SMOR, SON, TAM. [NEA, MTZ].

*Echinocereus* Engelm., 1848. 90/90. ALTN, ALTS, BCA, CABO, CAL, CGM, CPA, EVT, OAX, SMOC, SMOR, SON, TAM. [NEA, MTZ].

*Epiphyllum* Haw., 1812. 10/8. ALCH, BAL, CGM, CPA, EVT, OAX, PET, SMOC, SMOR, SMS, SOC, YUC. [NEO, MTZ].

*Epithelantha* Weber ex Britton & Rose, 1922. 6/4. ALTN, ALTS. [NEA, MTZ].

*Escobaria* Britton & Rose, 1923. 17/12. ALTN, ALTS, CGM, SMOC, SMOR, SON. [NEA, MTZ].

**Escontria* Rose, 1906. 1/1. BAL, CPA, OAX, SOC. [NEA, MTZ].

*Ferocactus* Britton & Rose, 1922. 35/35. ALTN, ALTS, BAL, BCA, CABO, CAL, CGM, CPA, EVT, OAX, SMOC, SMOR, SMS, SON, TAM. [NEA, MTZ].

**Geohintonia* Glass & F. Maurice, 1992. 1/1. ALTS, SMOR. [NEA, MTZ].

*Glandulicactus* Backeb., 1938. 4/2. ALTN, ALTS, TAM. [NEA, MTZ].

*Grusonia* F. Rchb. & K. Schum., 1896. 3/3. ALTN, ALTS, BCA, SON. [NEA].

**Lemaireocereus* Britton & Rose, 1909. 1/1. BAL, CGM, OAX. [NEA, MTZ].

**Leuchtenbergia* Hook., 1848. 1/1. ALTN, ALTS. [NEA, MTZ].

*Lophocereus* Britton & Rose, 1909. 5/5. ALTN, ALTS, BAL, BCA, CABO, CGM, CPA, EVT, OAX, SMOR, SMS, SON. [NEA, MTZ].

*Lophophora* J.M. Coult., 1894. 2/2. ALTN, ALTS, SMOC, SMOR, TAM. [NEA, MTZ].

*Mammillaria* Haw., 1812. 250/227. ALCH, ALTN, ALTS, BAL, BCA, CABO, CAL, CGM, CPA, EVT, OAX, PET, SMOC, SMOR, SMS, SOC, SON, TAM, YUC. [MTZ].

**Mammilloydia* Buxb., 1951. 1/1. ALTN, ALTS, SMOR. [NEA, MTZ].

*Myrtillocactus* Console, 1897. 4/3. ALTS, BAL, BCA, CABO, CGM, CPA, EVT, OAX, SMOC, SMOR, SMS. [NEA, MTZ].

*Neolloydia* Britton & Rose, 1922. 2/2. ALTN, ALTS, EVT, SMOR. [NEA, MTZ].

**Obregonia* Fric ex A. Berger, 1928. 1/1. ALTS. [NEA, MTZ].

*Opuntia* Mill., 1754. 150/97. ALCH, ALTN, ALTS, BAL, BCA, CABO, CAL, CGM, CPA, EVT, OAX, PET, SMOC, SMOR, SMS, SOC, SON, TAM, YUC. [MTZ].

**Ortegocactus* Alexander, 1961. 1/1. CPA. [NEA].

**Pachycereus* Britton & Rose, 1909. 5/5. ALTN, ALTS, BAL, BCA, CABO, CGM, CPA, OAX, SOC, SON. [NEA, MTZ].

**Pelecyphora* Ehrenb., 1843. 2/2. ALTN, ALTS. [NEA, MTZ].

*Peniocereus* Britton & Rose, 1909. 22/22. ALCH, ALTN, ALTS, BAL, BCA, CABO, CGM, CPA, EVT, OAX, SMOC, SMOR, SMS, SOC, SON. [MTZ].

*Pereskiopsis* Britton & Rose, 1907. 6/6. ALTS, BAL, BCA, CABO, CGM, CPA, EVT, OAX, SMOC, SMS, SOC, SON, YUC. [NEA, MTZ].

**Polaskia* Backeb., 1949. 2/2. OAX. [NEA, MTZ].

**Pterocereus* MacDougall & Miranda, 1954. 1/1. CGM, CPA, OAX, PET, SOC, YUC. [NEA].

*Sclerocactus* Britton & Rose, 1922. 1/1. ALTN, ALTS, TAM. [NEA, MTZ].

**Stenocactus* (K. Schum.) A. Berger, 1929. 19/19. ALTN, ALTS, BAL, EVT, OAX, SMOC, SMOR, SMS. [NEA, MTZ].

*Stenocereus* (A. Berger) Riccob., 1909. 23/22. ALCH, ALTN, ALTS, BAL, BCA, CABO, CGM, CPA, EVT, OAX, PET, SMOC, SMOR, SMS, SOC, SON, TAM, YUC. [MTZ].

**Strombocactus* Britton & Rose, 1922. 2/2. ALTS. [NEA, MTZ].

*Thelocactus* Britton & Rose, 1922. 16/16. ALTN, ALTS, OAX, SMOR, TAM. [NEA, MTZ].

**Turbinicarpus* Buxb. & Backeb., 1937. 24/24. ALTN, ALTS, EVT, SMOR. [NEA, MTZ].

**x Pachebergia* S. Arias & Terrazas, 2008. 1/1. BAL. [NEA, MTZ].

**x Pachgerocereus* Moran, 1962. 1/1. BCA. [NEA].

**Campanulaceae**

*Calcaratolobelia* Wilbur, 1997. 13/13. ALCH, ALTN, ALTS, BAL, CABO, CGM, CPA, EVT, OAX, SMOC, SMOR, SMS, SOC. [NEO, MTZ].

*Diastatea* Scheidw., 1841. 7/5. ALCH, ALTS, BAL, CGM, CPA, EVT, OAX, PET, SMOC, SMOR, SMS, SOC. [NEO, MTZ].

*Githopsis* Nutt., 1843. 4/2. CAL. [NEA].

*Heterotoma* Zucc., 1832. 2/2. EVT, SMOC, SMS, SOC. [NEO, MTZ].

*Hippobroma* G. Don, 1834. 1/1. BAL, CGM, CPA, OAX, PET, SOC, YUC. [MTZ].

*Nemacladus* Nutt., 1843. 18/10. BCA, CAL, SON. [NEA].

**Pseudonemacladus* McVaugh, 1943. 1/1. ALTS, SMOR. [NEA, MTZ].

*Triodanis* Raf. ex Greene, 1836. 6/3. ALTN, ALTS, BAL, BCA, CAL, CGM, CPA, EVT, OAX, SMOC, SMOR, SON, TAM. [MTZ].

**Wimmeranthus* Rzed., 2018. 1/1. BAL, OAX. [NEA, MTZ].

**Canellaceae**

*Canella* P. Browne, 1756. 1/1. PET, YUC. [NEO].

**Cannabaceae**

*Lozanella* Greenm., 1905. 2/1. ALCH, EVT, OAX, SMOR, SMS, SOC. [NEO, MTZ].

**Capparaceae**

*Caphexandra* Iltis & Cornejo, 2011. 1/1. ALCH, CGM, SMS, SOC. [NEA].

*Quadrella* (DC.) J. Presl, 1825. 23/12. ALTS, BAL, CGM, CPA, OAX, PET, SOC, TAM, YUC. [MTZ].

**Caprifoliaceae**

*Symphoricarpos* Ludw., 1760. 13/8. ALCH, ALTN, ALTS, CAL, EVT, OAX, SMOC, SMOR, SMS, SOC, SON. [MTZ].

**Caricaceae**

**Horovitzia* V.M. Badillo, 1993. 1/1. CGM, OAX. [NEA].

*Jarilla* Rusby, 1921. 4/4. ALCH, ALTS, BAL, CABO, CPA, EVT, SMOC, SMOR, SMS, SOC. [NEA, MTZ].

**Caryophyllaceae**

*Achyronychia* Torr. & A. Gray, 1868. 1/1. ALTS, BCA, CAL, EVT, OAX, SON. [NEA].

**Cerdia* Moç. & Sessé ex DC., 1828. 2/2. ALTS, EVT, SMOC, SMOR. [NEA, MTZ].

*Drymaria* Willd., 1819. 54/46. ALCH, ALTN, ALTS, BAL, BCA, CABO, CGM, CPA, EVT, OAX, SMOC, SMOR, SMS, SOC, SON, TAM, YUC. [MTZ].

**Pentacaena* Bartl., 1831. 1/1. EVT, OAX. [NEA].

*Scopulophila* M.E. Jones, 1908. 2/1. ALTS, EVT, OAX, SMOR. [NEA, MTZ].

**Celastraceae**

**Acanthothamnus* Brandegee, 1909. 1/1. ALTS, OAX. [NEA, MTZ].

*Canotia* Torr., 1857. 2/2. ALTN, SON. [NEA, MTZ].

*Gyminda* Sarg., 1891. 4/2. ALCH, CGM, CPA, OAX, PET, SMS, SOC, YUC. [NEO].

*Mortonia* A. Gray, 1852. 7/6. ALTN, ALTS, BAL, CGM, OAX, SMOC, SMOR, SMS, SON, TAM. [NEA, MTZ].

**Orthosphenia* Standl., 1923. 1/1. ALTS, SMOR. [NEA, MTZ].

*Paxistima* Raf., 1838. 2/1. SMOC, SMOR, SON. [NEA, MTZ].

*Quetzalia* Lundell, 1970. 11/6. ALCH, BAL, CGM, EVT, OAX, SMOR, SMS, SOC. [NEO, MTZ].

**Rzedowskia* Medrano, 1981. 1/1. ALTS. [NEA, MTZ].

*Schaefferia* Jacq., 1760. 15/8. ALCH, ALTN, ALTS, BAL, BCA, CABO, CGM, CPA, EVT, OAX, PET, SMOR, SMS, SOC, SON, TAM, YUC. [MTZ].

*Semialarium* N. Hallé, 1983. 2/1. ALCH, BAL, CGM, CPA, OAX, PET, SMOC, SOC, YUC. [NEO, MTZ].

*Wimmeria* Schltdl. & Cham., 1831. 24/14. ALCH, ALTN, ALTS, BAL, CGM, CPA, EVT, OAX, PET, SMOC, SMOR, SMS, SOC, TAM, YUC. [NEO, MTZ].

*Zinowiewia* Turcz., 1859. 9/7. ALCH, BAL, CGM, CPA, EVT, OAX, SMOR, SMS, SOC. [NEO, MTZ].

**Cleomaceae**

*Andinocleome* Iltis & Cochrane, 2014. 3/2. ALCH, CGM, CPA, EVT, OAX, SMOR, SMS, SOC. [MTZ].

*Atamisquea* Miers, 1826. 1/1. BCA, CABO, CPA, SON. [MTZ].

*Cleomella* DC., 1824. 4/4. ALTN, ALTS, CPA, EVT, OAX. [NEA, MTZ].

*Cleoserrata* Iltis, 2007. 2/2. ALCH, BAL, CGM, CPA, EVT, OAX, PET, SMOC, SMOR, SMS, SOC, YUC. [MTZ].

*Isomeris* Nutt., 1838. 4/2. BCA, SON. [NEA].

*Melidiscus* Raf., 1838. 2/2. ALCH, CGM, OAX, SMOR, SOC. [NEO, MTZ].

*Physostemon* Mart. & Zucc., 1824. 10/6. ALCH, BAL, BCA, CABO, CGM, CPA, EVT, OAX, PET, SMS, SOC, SON, YUC. [MTZ].

*Polanisia* Raf., 1819. 5/3. ALTN, ALTS, BAL, CGM, CPA, EVT, OAX, SMOC, SMOR, SMS, SON, TAM. [MTZ].

**Combretaceae**

*Conocarpus* L., 1753. 2/1. BAL, BCA, CABO, CGM, CPA, OAX, PET, SOC, TAM, YUC. [MTZ].

*Laguncularia* Gaertn. f., 1807. 1/1. ALTS, BAL, BCA, CABO, CGM, CPA, OAX, PET, SOC, SON, YUC. [MTZ].

**Commelinaceae**

*Callisia* Loefl., 1758. 20/16. ALCH, ALTN, ALTS, BAL, BCA, CABO, CGM, CPA, EVT, OAX, PET, SMOC, SMOR, SMS, SOC, TAM, YUC. [MTZ].

*Gibasis* Raf., 1837. 17/17. ALCH, ALTN, ALTS, BAL, BCA, CABO, CGM, CPA, EVT, OAX, PET, SMOC, SMOR, SMS, SOC, TAM, YUC. [NEO, MTZ].

**Gibasoides* D.R. Hunt, 1978. 1/1. OAX. [NEA, MTZ].

**Matudanthus* D.R. Hunt, 1978. 1/1. OAX, SMS. [NEA, MTZ].

**Thyrsanthemum* Pichon, 1946. 3/3. ALCH, ALTS, BAL, CGM, CPA, EVT, OAX, SMOC, SMOR, SMS, SOC. [NEA, MTZ].

*Tinantia* Scheidw., 1839. 13/9. ALCH, ALTN, ALTS, BAL, BCA, CABO, CGM, CPA, EVT, OAX, SMOC, SMOR, SMS, SOC, SON, TAM. [MTZ].

*Tradescantia* L., 1753. 70/48. ALCH, ALTN, ALTS, BAL, BCA, CABO, CGM, CPA, EVT, OAX, PET, SMOC, SMOR, SMS, SOC, SON, TAM, YUC. [MTZ].

*Tripogandra* Raf., 1837. 21/13. ALCH, ALTN, ALTS, BAL, CGM, CPA, EVT, OAX, PET, SMOC, SMOR, SMS, SOC, YUC. [NEO, MTZ].

*Weldenia* Schult. f., 1829. 1/1. EVT, OAX, SMOC, SMOR, SMS, SOC. [NEO, MTZ].

**Convolvulaceae**

*Aniseia* Choisy, 1834. 3/2. CGM, CPA, OAX, PET, SOC, YUC. [NEO].

*Iseia* O'Donell, 1953. 1/1. PET. [NEO].

*Itzaea* Standl. & Steyerm., 1944. 1/1. CGM, PET, SOC, YUC. [NEO].

*Odonellia* K.R. Robertson, 1982. 2/1. ALCH, CGM, CPA, OAX, PET, SMS, SOC. [NEO].

**Crassulaceae**

**Byrnesia* Rose, 1922. 1/1. CGM. [NEA, MTZ].

**Cremnophila* Rose, 1905. 3/3. BAL, EVT. [NEA, MTZ].

*Dudleya* Britton & Rose, 1903. 45/29. BCA, CABO, CAL, SON. [NEA].

*Echeveria* DC., 1828. 145/145. ALCH, ALTN, ALTS, BAL, CGM, CPA, EVT, OAX, SMOC, SMOR, SMS, SOC. [MTZ].

*Graptopetalum* Rose, 1911. 18/15. ALCH, ALTN, ALTS, BAL, CPA, EVT, OAX, SMOC, SMS, SOC, SON. [NEA, MTZ].

*Lenophyllum* Rose, 1904. 7/6. ALTN, ALTS, SMOR, TAM. [NEA, MTZ].

**Pachyphytum* Link, 1841. 19/19. ALTS, CGM, EVT, SMOC, SMOR. [NEA, MTZ].

**Tacitus* Moran, 1974. 1/1. SMOC. [NEA, MTZ].

**Thompsonella* Britton & Rose, 1909. 8/8. BAL, CPA, EVT, OAX, SMS. [NEA, MTZ].

*Villadia* Rose, 1903. 23/23. ALCH, ALTN, ALTS, BAL, CPA, EVT, OAX, SMOC, SMOR, SMS, SOC. [NEA, MTZ].

**Crossosomataceae**

*Crossosoma* Nutt., 1848. 3/3. ALTN, BCA, CAL, SON. [NEA].

**Velascoa* Calderón & Rzed., 1997. 1/1. SMOR. [NEA, MTZ].

**Cucurbitaceae**

**Apatzingania* Dieterle, 1974. 1/1. BAL. [NEA, MTZ].

*Brandegea* Cogn., 1890. 1/1. ALTS, BCA, CABO, SON. [NEA, MTZ].

**Chalema* Dieterle, 1980. 1/1. BAL, CPA. [NEA, MTZ].

*Cionosicyos* Benth. & Hook. f., 1867. 3/2. ALCH, BAL, CGM, CPA, EVT, OAX, PET, SMOR, SMS, SOC, YUC. [NEO, MTZ].

*Cucurbita* L., 1753. 20/19. ALCH, ALTN, ALTS, BAL, BCA, CABO, CAL, CGM, CPA, EVT, OAX, PET, SMOC, SMOR, SMS, SOC, SON, YUC. [MTZ].

*Cyclanthera* Schrad., 1831. 30/21. ALCH, ALTN, ALTS, BAL, BCA, CABO, CGM, CPA, EVT, OAX, SMOC, SMOR, SMS, SOC, SON. [MTZ].

**Dieterlea* E.J. Lott, 1986. 2/2. BAL, CPA. [NEA, MTZ].

*Doyerea* Grosourdy, 1864. 1/1. ALTS, BAL, CGM, CPA, OAX, PET, SOC, YUC. [NEO, MTZ].

*Echinopepon* Naudin, 1866. 19/11. ALCH, ALTN, ALTS, BAL, BCA, CABO, CGM, CPA, EVT, OAX, PET, SMOC, SMOR, SMS, SOC, SON. [MTZ].

*Ibervillea* Greene, 1895. 8/7. ALCH, ALTN, ALTS, BCA, CABO, CGM, CPA, OAX, PET, SMOC, SMOR, SMS, SOC, SON, TAM, YUC. [MTZ].

*Microsechium* Naudin, 1866. 3/2. ALCH, ALTS, BAL, CPA, EVT, OAX, SMOC, SMOR, SMS, SOC. [NEO, MTZ].

*Parasicyos* Dieterle, 1975. 2/2. OAX, SMOR, SMS, SOC. [NEO, MTZ].

**Peponopsis* Naudin, 1859. 1/1. CGM, CPA, SMOR. [NEA, MTZ].

*Polyclathra* Bertol., 1840. 2/2. ALCH, ALTS, BAL, BCA, CABO, CGM, CPA, EVT, OAX, PET, SMOC, SMS, SOC, YUC. [NEO, MTZ].

*Posadaea* Cogn., 1890. 1/1. CGM. [NEO].

*Psiguria* Neck. ex Arn., 1841. 6/3. ALCH, CGM, OAX, PET, SOC. [NEO, MTZ].

*Schizocarpum* Schrad., 1830. 11/9. ALCH, ALTS, BAL, CGM, CPA, EVT, OAX, SMOC, SMOR, SMS, SOC. [NEA, MTZ].

*Sechiopsis* Naudin, 1866. 5/5. ALCH, ALTS, BAL, CPA, EVT, OAX, SMOC, SOC. [NEO, MTZ].

*Sechium* P. Browne, 1756. 9/6. ALCH, ALTN, ALTS, BAL, CGM, CPA, EVT, OAX, PET, SMOC, SMOR, SMS, SOC. [MTZ].

*Sicydium* Schltdl., 1832. 8/5. ALCH, CGM, CPA, OAX, PET, SMOR, SMS, SOC, YUC. [NEO, MTZ].

*Sicyosperma* A. Gray, 1853. 1/1. ALTN, BCA, CABO, CPA, SMOC, SON. [NEA, MTZ].

*Tecunumania* Standl. & Steyerm., 1944. 1/1. CGM, CPA, OAX, SOC. [NEO].

*Tumamoca* Rose, 1912. 2/2. ALTN, ALTS, CPA, SON. [NEA, MTZ].

**Vaseyanthus* Cogn., 1891. 4/4. BCA, CABO, SON. [NEA, MTZ].

**Cyclanthaceae**

*Carludovica* Ruiz & Pav., 1794. 4/2. ALCH, CGM, OAX, SMOR, SOC. [NEO].

*Cyclanthus* Poit., 1822. 2/1. CGM. [NEO].

**Cymodoceaceae**

*Syringodium* Kütz., 1860. 2/1. TAM, YUC. [NEO].

**Cyperaceae**

*Amphiscirpus* Oteng-Yeb., 1974. 1/1. EVT. [MTZ].

**Cypringlea* M.T. Strong, 2003. 3/3. ALCH, ALTN, ALTS, OAX, SMOR, SMS. [NEA, MTZ].

**Karinia* Reznicek & McVaugh, 1993. 1/1. ALTN, ALTS, EVT, SMOC. [NEA, MTZ].

**Cyrillaceae**

*Cyrilla* L., 1767. 1/1. OAX. [NEO].

**Cytinaceae**

*Bdallophytum* Eichler, 1872. 5/3. ALCH, ALTS, BAL, CGM, CPA, EVT, OAX, SMOC, SMOR, SMS, SOC. [NEA, MTZ].

**Datiscaceae**

*Datisca* L., 1753. 2/1. CAL. [NEA].

**Dilleniaceae**

*Curatella* Loefl., 1758. 1/1. ALCH, BAL, CGM, CPA, EVT, OAX, PET, SMOC, SMOR, SMS, SOC, YUC. [NEO, MTZ].

**Ehretiaceae**

*Lennoa* Llave, 1824. 1/1. ALCH, ALTS, BAL, CGM, CPA, EVT, OAX, SMOC, SMOR, SMS, SOC. [NEO, MTZ].

*Lepidocordia* Ducke, 1925. 2/1. CPA, PET, SOC, YUC. [NEO].

*Pholisma* Nutt., 1844. 4/3. CAL, CPA, SON. [NEA, MTZ].

**Ericaceae**

*Andromeda* L., 1753. 2/1. EVT, OAX, SMOR. [NEO].

*Arbutus* L., 1753. 12/6. ALCH, ALTN, ALTS, BAL, BCA, CABO, CGM, CPA, EVT, OAX, SMOC, SMOR, SMS, SOC, SON, TAM. [MTZ].

*Chimaphila* Pursh, 1814. 5/3. ALCH, ALTN, ALTS, CAL, EVT, OAX, SMOC, SMOR, SMS, SOC, SON, TAM. [MTZ].

*Comarostaphylis* Zucc., 1837. 15/15. ALCH, ALTN, ALTS, BAL, CAL, CGM, CPA, EVT, OAX, SMOC, SMOR, SMS, SOC, TAM. [NEA, MTZ].

*Monotropa* L., 1753. 3/2. ALCH, ALTN, ALTS, BAL, CGM, CPA, EVT, OAX, SMOC, SMOR, SMS, SOC, SON. [MTZ].

*Ornithostaphylos* Small, 1914. 1/1. CAL. [NEA].

*Orthilia* Raf., 1840. 2/1. EVT, OAX, SMOC, SMOR, SOC. [MTZ].

*Pterospora* Nutt., 1818. 1/1. ALTN, ALTS, EVT, OAX, SMOC, SMOR, SON. [NEA, MTZ].

*Sarcodes* Torr., 1853. 1/1. CAL. [NEA].

*Xylococcus* Nutt., 1843. 1/1. BCA, CAL. [NEA].

**Eriocaulaceae**

*Tonina* Aubl., 1775. 1/1. SOC. [NEO].

**Euphorbiaceae**

*Adelia* L., 1759. 10/7. ALCH, ALTN, ALTS, BAL, BCA, CABO, CGM, CPA, EVT, OAX, PET, SMOR, SMS, SOC, SON, TAM, YUC. [MTZ].

*Cnidoscolus* Pohl, 1827. 50/28. ALCH, ALTN, ALTS, BAL, BCA, CABO, CGM, CPA, EVT, OAX, PET, SMOC, SMOR, SMS, SOC, SON, TAM, YUC. [MTZ].

*Dalembertia* Baill., 1858. 2/2. ALCH, ALTS, BAL, CPA, EVT, OAX, SMOC, SMOR, SMS, SOC. [NEO, MTZ].

**Enriquebeltrania* Rzed., 1979. 2/2. CPA, PET, YUC. [NEA].

*Garcia* Rohr, 1792. 2/2. CGM, CPA, OAX, PET, SOC, YUC. [NEO, MTZ].

*Hura* L., 1753. 1/1. ALCH, ALTS, BAL, CGM, CPA, EVT, OAX, PET, SMOC, SMOR, SMS, SOC, YUC. [NEO, MTZ].

*Pleradenophora* Esser, 2001. 6/5. ALCH, BAL, BCA, CABO, CGM, CPA, EVT, OAX, PET, SMOC, SON, YUC. [MTZ].

**Fabaceae**

*Acaciella* Britton & Rose, 1928. 18/18. ALCH, ALTN, ALTS, BAL, BCA, CABO, CGM, CPA, EVT, OAX, PET, SMOC, SMOR, SMS, SOC, SON, TAM, YUC. [MTZ].

*Acmispon* Raf., 1832. 24/12. ALTN, ALTS, BCA, CABO, CAL, EVT, OAX, SMOC, SMOR, SMS, SON. [MTZ].

*Apoplanesia* C. Presl, 1832. 2/1. BAL, CGM, CPA, OAX, PET, SOC, YUC. [NEO, MTZ].

*Ateleia* (Moç. & Sessé ex DC.) Benth., 1837. 27/15. ALCH, BAL, CGM, CPA, EVT, OAX, PET, SMOC, SMS, SOC, YUC. [NEO, MTZ].

*Barbieria* DC., 1825. 1/1. OAX. [NEO].

*Brongniartia* Kunth, 1824. 55/51. ALCH, ALTN, ALTS, BAL, BCA, CABO, CGM, CPA, EVT, OAX, SMOC, SMOR, SMS, SOC, SON. [MTZ].

**Calliandropsis* H.M. Hern. & P. Guinet, 1990. 1/1. OAX. [NEA, MTZ].

*Cologania* Kunth, 1824. 16/16. ALCH, ALTN, ALTS, BAL, CGM, CPA, EVT, OAX, SMOC, SMOR, SMS, SOC, SON, TAM. [MTZ].

**Conzattia* Rose, 1909. 2/2. ALCH, ALTN, ALTS, BAL, CABO, CGM, CPA, EVT, OAX, PET, SMOC, SMOR, SMS, SOC, 0. [NEA, MTZ].

*Coulteria* Kunth, 1824. 6/5. ALTN, ALTS, BAL, CGM, CPA, OAX, PET, SOC, SON, TAM, YUC. [NEO, MTZ].

*Coursetia* DC., 1825. 38/24. ALCH, ALTN, ALTS, BAL, BCA, CABO, CGM, CPA, EVT, OAX, PET, SMOC, SMOR, SMS, SOC, SON, TAM, YUC. [MTZ].

*Cymbosema* Benth., 1840. 2/1. CPA, SOC. [NEO].

*Dalea* Juss., 1789. 184/184. ALCH, ALTN, ALTS, BAL, BCA, CABO, CAL, CGM, CPA, EVT, OAX, PET, SMOC, SMOR, SMS, SOC, SON, TAM, YUC. [MTZ].

*Dermatophyllum* Scheele, 1848. 5/4. ALTN, ALTS, BAL, CGM, EVT, OAX, SMOC, SMOR, TAM. [NEA, MTZ].

*Desmanthus* Willd., 1806. 24/18. ALCH, ALTN, ALTS, BAL, BCA, CABO, CGM, CPA, EVT, OAX, PET, SMOC, SMOR, SMS, SOC, SON, TAM, YUC. [MTZ].

*Desmodium* Desv., 1813. 199/121. ALCH, ALTN, ALTS, BAL, BCA, CABO, CGM, CPA, EVT, OAX, PET, SMOC, SMOR, SMS, SOC, SON, TAM, YUC. [MTZ].

*Diphysa* Jacq., 1760. 19/19. ALCH, ALTN, ALTS, BAL, BCA, CABO, CGM, CPA, EVT, OAX, PET, SMOC, SMOR, SMS, SOC, SON, TAM, YUC. [MTZ].

*Ebenopsis* Britton & Rose, 1928. 2/2. ALTN, ALTS, BCA, CABO, CGM, CPA, PET, SMOC, SMOR, SON, TAM, YUC. [NEA, MTZ].

*Errazurizia* Phil., 1872. 4/2. BCA, SON. [NEA].

*Erythrostemon* Klotzsch, 1844. 33/23. ALCH, ALTN, ALTS, BAL, BCA, CABO, CAL, CGM, CPA, EVT, OAX, PET, SMOC, SMOR, SMS, SOC, SON, TAM, YUC. [MTZ].

*Eysenhardtia* Kunth, 1824. 13/13. ALCH, ALTN, ALTS, BAL, BCA, CGM, CPA, EVT, OAX, SMOC, SMOR, SMS, SOC, SON, TAM. [NEA, MTZ].

*Genistidium* I.M. Johnst., 1941. 1/1. ALTN, ALTS. [NEA].

*Gliricidia* Kunth, 1824. 4/3. ALCH, ALTS, BAL, CGM, CPA, EVT, OAX, PET, SMOC, SMOR, SMS, SOC, YUC. [NEO, MTZ].

**Guinetia* L. Rico & M. Sousa, 1999. 1/1. CPA. [NEA].

*Haematoxylum* L., 1753. 5/4. ALCH, ALTN, ALTS, BAL, BCA, CABO, CGM, CPA, EVT, OAX, PET, SMOC, SMS, SOC, SON, TAM, YUC. [NEO, MTZ].

*Havardia* Small, 1901. 5/5. ALCH, ALTN, ALTS, BAL, BCA, CABO, CGM, CPA, OAX, PET, SMOC, SMOR, SMS, SOC, SON, TAM, YUC. [NEA, MTZ].

*Helicotropis* A. Delgado, 2011. 3/2. ALCH, BAL, CGM, CPA, EVT, OAX, SMOC, SMOR, SMS, SOC. [NEO, MTZ].

**Hesperothamnus* Brandegee, 1919. 4/4. ALTS, BCA, CABO, OAX. [NEA, MTZ].

**Heteroflorum* M. Sousa, 2005. 1/1. BAL, CPA. [NEA, MTZ].

*Hoita* Rydb., 1919. 3/2. CAL. [NEA].

*Hybosema* Harms, 1923. 2/2. ALCH, ALTS, BAL, CGM, CPA, OAX, SMOR, SMS, SOC. [NEA, MTZ].

*Lennea* Klotzsch, 1842. 5/4. ALCH, CGM, CPA, OAX, PET, SOC, YUC. [NEA, MTZ].

*Leptospron* (Benth.) A. Delgado, 2011. 2/2. ALCH, BAL, CGM, CPA, EVT, OAX, PET, SMOC, SMOR, SMS, SOC. [MTZ].

*Leucaena* Benth., 1842. 25/25. ALCH, ALTN, ALTS, BAL, BCA, CABO, CGM, CPA, EVT, OAX, PET, SMOC, SMOR, SMS, SOC, SON, TAM, YUC. [MTZ].

*Lonchocarpus* Kunth, 1824. 161/95. ALCH, ALTN, ALTS, BAL, CGM, CPA, EVT, OAX, PET, SMOC, SMOR, SMS, SOC, TAM, YUC. [MTZ].

*Lysiloma* Benth., 1844. 9/7. ALCH, ALTN, ALTS, BAL, BCA, CABO, CGM, CPA, EVT, OAX, PET, SMOC, SMOR, SMS, SOC, SON, TAM, YUC. [MTZ].

*Marina* Liebm., 1854. 42/42. ALCH, ALTN, ALTS, BAL, BCA, CABO, CAL, CGM, CPA, EVT, OAX, SMOC, SMOR, SMS, SOC, SON. [MTZ].

*Mariosousa* Seigler & Ebinger, 2006. 13/13. ALCH, ALTN, ALTS, BAL, BCA, CABO, CGM, CPA, EVT, OAX, PET, SMOC, SMOR, SMS, SOC, SON, TAM, YUC. [NEA, MTZ].

*Microlobius* C. Presl, 1845. 1/1. BAL, CPA, OAX, SOC. [NEO, MTZ].

*Myrospermum* Jacq., 1760. 3/2. BAL, CGM, CPA, OAX, SOC. [MTZ].

*Myroxylon* L. f., 1882. 2/2. ALCH, BAL, CGM, CPA, OAX, PET, SMOC, SOC, YUC. [NEO, MTZ].

*Nissolia* Jacq., 1760. 15/14. ALCH, ALTN, ALTS, BAL, BCA, CABO, CGM, CPA, EVT, OAX, PET, SMOC, SMOR, SMS, SOC, SON, YUC. [MTZ].

*Olneya* A. Gray, 1854. 1/1. BCA, CABO, CAL, CPA, SON. [NEA, MTZ].

*Orobus* L., 1753. 1/1. [NEA].

*Oxyrhynchus* Brandegee, 1912. 4/3. ALCH, CGM, SMOR, TAM. [MTZ].

*Pachecoa* Standl. & Steyerm., 1943. 1/1. BAL, CGM, CPA, OAX. [NEO, MTZ].

**Painteria* Britton & Rose, 1928. 3/3. ALTN, ALTS, BAL, EVT, OAX, SMOC, SMOR, SMS. [NEA, MTZ].

*Parkinsonia* L., 1753. 11/8. ALCH, ALTN, ALTS, BAL, BCA, CABO, CAL, CGM, CPA, EVT, OAX, PET, SMOC, SMOR, SMS, SOC, SON, TAM, YUC. [MTZ].

*Peteria* A. Gray, 1852. 4/3. ALTN, ALTS, EVT, SMOC, SMOR. [NEA, MTZ].

*Phaseolus* L., 1753. 72/72. ALCH, ALTN, ALTS, BAL, BCA, CABO, CGM, CPA, EVT, OAX, PET, SMOC, SMOR, SMS, SOC, SON, TAM, YUC. [MTZ].

*Pickeringia* Nutt. ex Torr. & A. Gray, 1840. 1/1. CAL. [NEA].

*Piscidia* L., 1759. 7/5. ALCH, ALTN, ALTS, BAL, CGM, CPA, EVT, OAX, PET, SMOC, SMOR, SMS, SOC, SON, YUC. [MTZ].

*Poeppigia* C. Presl, 1830. 1/1. BAL, CGM, CPA, OAX, SOC. [NEO, MTZ].

*Pomaria* Cav., 1799. 16/8. ALTN, ALTS, CGM, CPA, EVT, SMOC, SMOR. [MTZ].

*Psoralidium* Rydb., 1919. 1/1. ALTN, SON. [NEA].

*Psorothamnus* Rydb., 1919. 10/6. ALTN, BCA, CABO, CAL, SON. [NEA, MTZ].

*Ramirezella* Rose, 1903. 9/9. ALCH, ALTS, BAL, CGM, CPA, EVT, SMOC, SMOR, SMS, SOC. [NEO, MTZ].

*Schizolobium* Vogel, 1837. 1/1. ALCH, CGM, CPA, OAX, PET, SOC, YUC. [NEO, MTZ].

*Sphinctospermum* Rose, 1906. 1/1. ALTS, BAL, BCA, CABO, CPA, SON. [NEA, MTZ].

*Sphinga* Barneby & J.W. Grimes, 1996. 3/2. ALCH, ALTS, BAL, CGM, CPA, EVT, OAX, PET, SMOC, SMOR, SMS, SOC, YUC. [NEO, MTZ].

*Strophostyles* Elliott, 1823. 3/2. SMOR. [MTZ].

*Styphnolobium* Schott ex Endl., 1831. 9/5. BAL, CGM, CPA, OAX. [MTZ].

*Tara* Molina, 1810. 3/2. ALCH, ALTS, BAL, CGM, CPA, EVT, OAX, PET, SMOC, SMS, SOC, YUC. [NEO, MTZ].

*Zapoteca* H.M. Hern., 1989. 21/18. ALCH, ALTN, ALTS, BAL, BCA, CABO, CGM, CPA, EVT, OAX, PET, SMOC, SMOR, SMS, SOC, SON, YUC. [MTZ].

**Fouquieriaceae**

*Fouquieria* Kunth, 1819. 13/13. ALTN, ALTS, BAL, BCA, CABO, CAL, CGM, CPA, EVT, OAX, SMOC, SMOR, SMS, SOC, SON, TAM. [NEA, MTZ].

**Garryaceae**

*Garrya* Douglas, 1834. 15/13. ALCH, ALTN, ALTS, BAL, BCA, CABO, CAL, CGM, CPA, EVT, OAX, SMOC, SMOR, SMS, SOC, SON, TAM. [MTZ].

**Gentianaceae**

*Eustoma* Salisb., 1806. 3/3. ALCH, ALTN, ALTS, BAL, BCA, CABO, CAL, CGM, CPA, EVT, OAX, PET, SMOC, SMOR, SMS, SOC, SON, TAM, YUC. [MTZ].

**Geniostemon* Engelm. & A. Gray, 1881. 4/4. ALTS, SMOR, TAM. [NEA, MTZ].

*Gyrandra* Griseb., 1845. 8/5. ALTS, BAL, CPA, EVT, OAX, SMOC, SMOR, SMS. [NEO, MTZ].

*Zeltnera* G. Mans., 2004. 26/13. ALCH, ALTN, ALTS, BAL, BCA, CABO, CAL, CGM, CPA, EVT, OAX, PET, SMOC, SMOR, SMS, SOC, SON, TAM, YUC. [MTZ].

**Geraniaceae**

*California* Aldasoro, C. Navarro, P. Vargas, L. Sáez & Aedo, 2002. 1/1. CAL. [NEA].

**Gesneriaceae**

*Achimenes* Pers., 1806. 30/26. ALCH, ALTS, BAL, CGM, CPA, EVT, OAX, PET, SMOC, SMOR, SMS, SOC. [NEO, MTZ].

*Alsobia* Hanst., 1854. 4/2. ALCH, CPA, SOC. [NEO].

*Amalophyllon* Brandegee, 1914. 2/2. ALCH, CGM, CPA, SOC. [NEO].

*Cobananthus* Wiehler, 1977. 1/1. CGM. [NEO].

**Eucodonia* Hanst., 1854. 2/2. ALCH, BAL, CGM, CPA, EVT, OAX, SMS, SOC. [NEA, MTZ].

*Moussonia* Regel, 1847. 18/12. ALCH, ALTS, BAL, CGM, CPA, EVT, OAX, SMOC, SMOR, SMS, SOC. [NEO, MTZ].

*Neomortonia* Wiehler, 1975. 1/1. SOC. [NEO].

*Niphaea* Lindl., 1841. 3/3. EVT, SMOC. [NEA, MTZ].

*Phinaea* Benth., 1871. 3/2. BAL, CPA, EVT, SMS. [NEO, MTZ].

**Smithiantha* Kuntze, 1891. 6/6. ALCH, CGM, EVT, OAX, SMOR, SOC. [NEA, MTZ].

*Solenophora* Benth., 1840. 19/12. ALCH, CGM, CPA, EVT, OAX, PET, SMOR, SMS, SOC. [NEO].

**Guamatelaceae**

*Guamatela* Donn. Sm., 1914. 1/1. CGM, OAX. [NEO].

**Haemodoraceae**

*Xiphidium* Aubl., 1775. 2/1. ALCH, CGM, CPA, OAX, SMOR, SMS, SOC. [NEO, MTZ].

**Haloragaceae**

*Proserpinaca* L., 1759. 2/2. ALCH, OAX, SOC. [NEO].

**Hamamelidaceae**

*Matudaea* Lundell, 1940. 2/2. ALCH, BAL, CPA, EVT, OAX, SMOR, SMS, SOC. [NEO, MTZ].

**Hernandiaceae**

*Gyrocarpus* Jacq., 1763. 5/3. ALCH, ALTS, BAL, CGM, CPA, EVT, OAX, PET, SMOC, SMOR, SMS, SOC, YUC. [MTZ].

**Hydrangeaceae**

*Fendlera* Engelm. & A. Gray, 1852. 5/4. ALTN, ALTS, SMOC, SMOR, SON. [NEA, MTZ].

*Fendlerella* Heller, 1898. 4/4. ALTN, ALTS, SMOC, SMOR, SON. [NEA, MTZ].

*Jamesia* Torr. & A. Gray, 1840. 2/1. SMOC, SMOR. [NEA, MTZ].

*Philadelphus* L., 1753. 25/21. ALCH, ALTN, ALTS, BCA, EVT, OAX, SMOC, SMOR, SMS, SOC, SON. [MTZ].

**Hydrocharitaceae**

*Thalassia* Banks & Sol. ex K.D. Koenig, 1805. 2/1. TAM, YUC. [NEA].

**Hydrophyllaceae**

*Emmenanthe* Benth., 1835. 1/1. BCA, CAL. [NEA].

*Eucrypta* Nutt., 1848. 3/3. ALTN, BCA, CAL, CPA, SON. [NEA, MTZ].

*Pholistoma* Lilja, 1839. 3/3. BCA, CAL, SON. [NEA].

**Hypericaceae**

*Thornea* Breedlove & E.M. McClint., 1976. 2/2. ALCH, SOC. [NEA].

**Iridaceae**

**Ainea* Ravenna, 1979. 1/1. OAX, SMS. [NEA, MTZ].

*Alophia* Herb., 1840. 5/4. ALCH, ALTN, ALTS, CGM, CPA, OAX, PET, SMOR, SOC, TAM, YUC. [MTZ].

**Colima* (Ravenna) Aarón Rodr. & Ortiz-Catedral, 2003. 1/1. BAL, CPA, SMS. [NEA, MTZ].

*Eleutherine* Herb., 1843. 3/2. ALCH, BAL, CGM, CPA, EVT, OAX, SMOR, SMS. [NEO, MTZ].

**Fosteria* Molseed, 1968. 1/1. OAX, SMS. [NEA, MTZ].

*Sisyrinchium* Mill., 1754. 80/42. ALCH, ALTN, ALTS, BAL, BCA, CABO, CAL, CGM, CPA, EVT, OAX, SMOC, SMOR, SMS, SOC, SON, TAM. [MTZ].

*Tigridia* Juss., 1789. 50/50. ALCH, ALTN, ALTS, BAL, CGM, CPA, EVT, OAX, SMOC, SMOR, SMS, SOC. [NEO, MTZ].

**Iteaceae**

**Pterostemon* Schauer, 1847. 3/3. ALTS, EVT, OAX, SMOR, SMS. [NEA, MTZ].

**Koeberliniaceae**

*Koeberlinia* Zucc., 1832. 2/1. ALTN, ALTS, BCA, CGM, CPA, OAX, SON, TAM. [MTZ].

**Krameriaceae**

*Krameria* Loefl., 1762. 18/9. ALCH, ALTN, ALTS, BAL, BCA, CABO, CAL, CGM, CPA, EVT, OAX, SMOC, SMOR, SMS, SOC, SON, TAM. [MTZ].

**Lamiaceae**

*Agastache* J. Clayton ex Gronov., 1762. 21/18. ALTN, ALTS, EVT, OAX, SMOC, SMOR, SON. [NEA, MTZ].

*Asterohyptis* Epling, 1932. 4/4. ALCH, ALTN, ALTS, BAL, CGM, CPA, EVT, OAX, SMOC, SMOR, SMS, SOC, SON. [NEA, MTZ].

*Catoferia* (Benth.) Benth., 1876. 4/3. ALCH, BAL, CGM, CPA, SOC. [NEA].

*Hedeoma* Juss., 1821. 46/34. ALCH, ALTN, ALTS, BCA, EVT, OAX, SMOC, SMOR, SON, TAM. [MTZ].

*Poliomintha* A. Gray, 1870. 8/8. ALTN, ALTS, SMOC, SMOR. [NEA, MTZ].

*Warnockia* M.W. Turner, 1996. 1/1. ALTN, ALTS. [NEA].

**Lauraceae**

*Damburneya* Raf., 1838. 20/13. ALCH, ALTS, BAL, CGM, CPA, EVT, OAX, PET, SMOC, SMOR, SMS, SOC, TAM, YUC. [MTZ].

**Mocinnodaphne* Lorea Hern., 1995. 1/1. OAX, SMS. [NEA].

*Umbellularia* Nutt., 1842. 1/1. CAL. [NEA].

**Loasaceae**

*Cevallia* Lag., 1805. 1/1. ALTN, ALTS, EVT, SMOC, SMOR, SON, TAM. [NEA, MTZ].

*Eucnide* Zucc., 1844. 14/14. ALCH, ALTN, ALTS, BAL, BCA, CABO, CAL, CGM, CPA, EVT, OAX, SMOC, SMOR, SMS, SOC, SON. [MTZ].

*Gronovia* L., 1753. 2/2. ALCH, ALTN, ALTS, BAL, CGM, CPA, EVT, OAX, PET, SMOC, SMOR, SMS, SOC, SON, YUC. [NEO, MTZ].

*Klaprothia* Kunth, 1823. 2/1. ALCH, CPA, EVT, SMOC, SMS, SOC. [NEO, MTZ].

*Mentzelia* L., 1753. 49/31. ALCH, ALTN, ALTS, BAL, BCA, CABO, CAL, CGM, CPA, EVT, OAX, PET, SMOC, SMOR, SMS, SOC, SON, TAM, YUC. [MTZ].

*Petalonyx* A. Gray, 1855. 4/3. ALTN, BCA, CAL, SON. [NEA, MTZ].

**Schismocarpus* S.F. Blake, 1918. 2/2. CPA, OAX, SOC. [NEA].

**Loranthaceae**

*Cladocolea* Tiegh., 1895. 41/22. BAL, CGM, CPA, EVT, OAX, SMOC, SMS, SOC. [NEO, MTZ].

**Lythraceae**

*Adenaria* Kunth, 1823. 1/1. ALCH, BAL, CGM, CPA, OAX, SMS, SOC. [NEO, MTZ].

**Malpighiaceae**

*Aspicarpa* Rich., 1815. 9/5. ALTN, ALTS, EVT, SMOC. [MTZ].

**Calcicola* W.R. Anderson & C. Davis, 2007. 2/2. ALTN, ALTS, BAL, CPA, OAX, SMOC, SMOR, SON. [NEA, MTZ].

*Callaeum* Small, 1910. 11/7. ALCH, ALTN, ALTS, BAL, BCA, CABO, CGM, CPA, EVT, OAX, PET, SMOC, SMOR, SMS, SOC, SON, TAM. [MTZ].

*Cottsia* Dubard & Dop, 1908. 3/3. ALTN, ALTS, BCA, CABO, CPA, SMOC, SON. [NEA, MTZ].

**Echinopterys* Juss., 1843. 2/2. ALTN, ALTS, BAL, CPA, OAX, SON. [NEA, MTZ].

*Galphimia* Cav., 1799. 26/21. ALCH, ALTN, ALTS, BAL, BCA, CABO, CGM, CPA, EVT, OAX, SMOC, SMOR, SMS, SOC, SON. [MTZ].

*Gaudichaudia* Kunth, 1822. 30/24. ALCH, ALTN, ALTS, BAL, CGM, CPA, EVT, OAX, PET, SMOC, SMOR, SMS, SOC, SON, YUC. [MTZ].

**Lasiocarpus* Liebm., 1854. 2/2. BAL, CGM, CPA, EVT, OAX, SMOC, SMS, SOC. [NEA, MTZ].

*Psychopterys* W.R. Anderson & S. Corso, 2007. 8/6. ALCH, BAL, CGM, CPA, EVT, OAX, PET, SMS, SOC. [NEO, MTZ].

**Malvaceae**

*Allosidastrum* (Hochr.) Krapov., Fryxell & D.M. Bates, 1988. 4/3. ALCH, ALTS, BAL, CGM, CPA, EVT, OAX, PET, SMOC, SMOR, SMS, SOC. [NEO, MTZ].

*Allowissadula* Bates, 1978. 9/9. ALTN, ALTS, BAL, CGM, CPA, OAX, PET, TAM, YUC. [NEA, MTZ].

*Anoda* Cav., 1785. 24/23. ALCH, ALTN, ALTS, BAL, BCA, CABO, CGM, CPA, EVT, OAX, PET, SMOC, SMOR, SMS, SOC, SON, TAM, YUC. [MTZ].

**Anotea* (DC.) Kunth, 1846. 1/1. CPA. [NEA].

*Bakeridesia* Hochr., 1913. 15/15. ALCH, ALTS, BAL, CGM, CPA, OAX, PET, SMOR, SMS, SOC, SON, TAM, YUC. [NEO, MTZ].

**Bastardiastrum* (Rose) Bates, 1978. 8/8. ALCH, BAL, CPA, OAX, SMOC, SMS, SOC, SON. [NEA, MTZ].

*Batesimalva* Fryxell, 1976. 5/3. ALTN, ALTS, CGM, SMOR. [NEA, MTZ].

*Bernoullia* Oliver, 1873. 3/2. ALCH, BAL, CGM, CPA, EVT, OAX, PET, SMOC, SMOR, SMS, SOC, YUC. [NEO, MTZ].

*Billieturnera* Fryxell, 1982. 1/1. TAM. [NEA].

*Briquetia* Hochr., 1902. 5/3. ALCH, BAL, CGM, CPA, EVT, OAX, SMOC, SMS, SOC, SON. [NEO, MTZ].

*Chiranthodendron* Larreat., 1805. 1/1. ALCH, EVT, OAX, SMS, SOC. [NEA, MTZ].

**Dendrosida* Fryxell, 1971. 6/6. ALCH, BAL, CPA, SMS, SOC. [NEA, MTZ].

*Dirhamphis* Krapov., 1970. 2/1. BAL, CPA. [NEO].

*Eremalche* Greene, 1906. 3/2. BCA, CAL, SON. [NEA].

*Fremontodendron* Coville, 1893. 2/2. CAL. [NEA].

*Fryxellia* Bates, 1974. 1/1. ALTN. [NEA].

*Hampea* Schltdl., 1837. 20/11. ALCH, BAL, CGM, CPA, EVT, OAX, PET, SMOR, SOC, YUC. [MTZ].

*Heliocarpus* L., 1753. 11/11. ALCH, ALTN, ALTS, BAL, CGM, CPA, EVT, OAX, PET, SMOC, SMOR, SMS, SOC, SON, YUC. [NEO, MTZ].

*Hochreutinera* Krapov., 1970. 2/1. BAL, CGM, CPA, EVT, OAX, SMOC, SMOR, SMS, SOC. [NEO, MTZ].

*Horsfordia* A. Gray, 1887. 4/4. ALTS, BCA, CABO, CAL, CPA, SON. [NEA, MTZ].

*Kearnemalvastrum* Bates, 1967. 2/2. ALCH, ALTS, BAL, CGM, CPA, EVT, OAX, SMOR, SMS, SOC. [NEO, MTZ].

*Lopimia* Martius, 1823. 2/1. ALCH, CGM, CPA, OAX, SOC. [NEO, MTZ].

*Malvaviscus* Fabr., 1759. 11/7. ALCH, ALTN, ALTS, BAL, CGM, CPA, EVT, OAX, PET, SMOC, SMOR, SMS, SOC, TAM, YUC. [MTZ].

*Malvella* Jaub. & Spach, 1855. 4/3. ALTN, ALTS, BCA, CAL, CPA, OAX, SON. [MTZ].

*Meximalva* Fryxell, 1976. 2/2. ALTN, ALTS, TAM. [NEA, MTZ].

*Mortoniodendron* Standl. & Steyerm., 1938. 16/8. ALCH, CGM, OAX, PET, SMOR, SOC. [NEO].

*Neobrittonia* Hochr., 1905. 1/1. ALCH, EVT, SMS, SOC. [NEO, MTZ].

*Ochroma* Sw., 1788. 1/1. ALCH, CGM, OAX, PET, SOC. [NEO].

*Periptera* DC., 1824. 5/5. ALTS, BAL, CGM, CPA, EVT, OAX, SMOC, SMOR, SMS. [NEO, MTZ].

*Phymosia* Ham., 1825. 8/7. ALCH, ALTS, BAL, CGM, CPA, EVT, OAX, SMOC, SMOR, SMS, SOC. [NEO, MTZ].

**Physodium* C. Presl, 1836. 2/2. ALTS, BAL, CPA, OAX. [NEA, MTZ].

*Rhynchosida* Fryxell, 1978. 2/1. ALTN, ALTS, SON, TAM. [MTZ].

*Robinsonella* Rose & E.G. Baker, 1897. 16/15. ALCH, ALTS, BAL, CGM, CPA, EVT, OAX, SMOC, SMOR, SMS, SOC, TAM. [NEO, MTZ].

*Sidastrum* Baker f., 1892. 10/5. ALCH, CABO, CGM, CPA, OAX, SMOC, SMOR, SOC, TAM. [MTZ].

*Sphaeralcea* A. St.-Hil., 1827. 40/22. ALCH, ALTN, ALTS, BCA, CABO, CAL, CGM, CPA, EVT, OAX, SMOC, SMOR, SOC, SON, TAM. [MTZ].

**Martyniaceae**

*Martynia* L., 1753. 2/1. ALCH, ALTS, BAL, CGM, CPA, EVT, OAX, PET, SMOC, SMOR, SMS, SOC, YUC. [NEO, MTZ].

*Proboscidea* Adans., 1763. 8/8. ALTN, ALTS, BAL, BCA, CABO, CAL, CGM, CPA, EVT, OAX, SMOC, SMOR, SMS, SON, TAM. [MTZ].

**Melanthiaceae**

*Anticlea* Kunth, 1843. 11/6. ALTN, ALTS, CAL, EVT, OAX, SMOC, SMOR, SMS, SOC. [MTZ].

*Schoenocaulon* A. Gray, 1837. 29/29. ALCH, ALTN, ALTS, BAL, CGM, CPA, EVT, OAX, PET, SMOC, SMOR, SMS, SOC, TAM. [MTZ].

*Zigadenus* Michx., 1803. 2/2. CAL, SON. [NEA].

**Melastomataceae**

*Arthrostemma* Ruiz & Pav., 1802. 4/4. ALCH, BAL, CGM, CPA, EVT, OAX, PET, SMOC, SMOR, SMS, SOC. [MTZ].

*Centradenia* G. Don, 1832. 4/3. ALCH, CGM, CPA, EVT, OAX, SMOR, SMS, SOC. [MTZ].

*Heterocentron* Hook. & Arn., 1840. 11/9. ALCH, ALTS, BAL, CGM, CPA, EVT, OAX, SMOC, SMOR, SMS, SOC. [MTZ].

*Schwackaea* Cogn., 1888. 1/1. SOC. [NEO].

*Stanmarkia* Almeda, 1993. 2/1. ALCH, SOC. [NEA].

**Meliaceae**

*Swietenia* Jacq., 1760. 3/2. ALCH, ALTS, BAL, CGM, CPA, EVT, OAX, PET, SMOC, SMOR, SMS, SOC, YUC. [MTZ].

**Menispermaceae**

*Menispermum* L., 1753. 2/1. SMOR. [MTZ].

**Metteniusaceae**

*Calatola* Standl., 1923. 6/4. ALCH, CGM, CPA, EVT, OAX, SMOR, SMS, SOC. [NEO, MTZ].

*Oecopetalum* Greenm. & C.H. Thomps., 1915. 2/2. ALCH, CGM, EVT, OAX, PET, SMOR, SOC. [NEO, MTZ].

**Mitrastemonaceae**

*Mitrastemon* Makino, 1911. 2/1. ALCH, BAL, CPA, SMS, SOC. [NEO, ZTM].

**Montiaceae**

*Phemeranthus* Raf., 1814. 21/11. ALTN, ALTS, EVT, OAX, SMOC, SMOR, SON. [MTZ].

**Moraceae**

*Clarisia* Ruiz & Pav., 1794. 4/2. ALCH, CGM, CPA, OAX, PET, SOC. [NEO, MTZ].

*Poulsenia* Eggers, 1898. 1/1. ALCH, CGM, CPA, OAX, PET, SOC. [NEO].

*Trophis* L., 1759. 10/5. ALCH, ALTN, ALTS, BAL, CGM, CPA, EVT, OAX, PET, SMOC, SMOR, SMS, SOC, TAM, YUC. [NEO, MTZ].

**Muntingiaceae**

*Muntingia* L., 1753. 1/1. ALCH, ALTN, ALTS, BAL, CGM, CPA, EVT, OAX, PET, SMOC, SMOR, SMS, SOC, YUC. [NEO, MTZ].

**Myrtaceae**

*Chamguava* Landrum, 1991. 3/2. ALCH, CGM, CPA, PET, SOC. [NEO].

**Namaceae**

*Nama* L., 1759. 53/47. ALCH, ALTN, ALTS, BAL, BCA, CABO, CAL, CGM, CPA, EVT, OAX, PET, SMOC, SMOR, SMS, SOC, SON, TAM, YUC. [MTZ].

*Turricula* J.F. Macbr., 1917. 1/1. CAL. [NEA].

**Nelumbonaceae**

*Nelumbo* Adans., 1763. 2/1. TAM. [MTZ].

**Nyctaginaceae**

*Abronia* Juss. ex Lam., 1791. 20/10. ALTN, BCA, CABO, CAL, CPA, SON. [NEA, MTZ].

*Acleisanthes* A. Gray, 1853. 16/12. ALTN, ALTS, SMOC, SMOR, SON, TAM. [MTZ].

*Allionia* L., 1759. 4/4. ALTN, ALTS, BAL, BCA, CABO, CAL, CGM, CPA, EVT, OAX, SMOC, SMOR, SON, TAM. [MTZ].

*Anulocaulis* Standl., 1909. 5/5. ALTN, ALTS. [NEA].

*Cryptocarpus* Kunth, 1818. 1/1. CPA. [NEO].

*Cyphomeris* Standl., 1911. 2/2. ALTN, ALTS, CGM, OAX, SMOR. [NEA, MTZ].

*Grajalesia* Miranda, 1951. 2/1. CGM, CPA, SOC. [NEO].

*Mirabilis* L., 1753. 60/38. ALCH, ALTN, ALTS, BAL, BCA, CABO, CAL, CGM, CPA, EVT, OAX, PET, SMOC, SMOR, SMS, SOC, SON, TAM, YUC. [MTZ].

*Nyctaginia* Choisy, 1849. 1/1. ALTN, ALTS, TAM. [NEA].

*Okenia* Schltdl. & Cham., 1830. 4/3. BAL, CGM, CPA, EVT, PET, SMOC, SMOR, SMS, YUC. [NEA, MTZ].

*Pisoniella* (Heimerl) Standl., 1911. 2/1. ALTS, BAL, CGM, CPA, EVT, OAX, SMOR, SMS, SOC. [NEO, MTZ].

*Salpianthus* Humb. & Bonpl., 1808. 5/5. ALCH, ALTS, BAL, CGM, CPA, EVT, OAX, SMOC, SMS, SOC. [NEO, MTZ].

**Oleaceae**

*Forestiera* Poir., 1811. 20/12. ALCH, ALTN, ALTS, BAL, BCA, CABO, CGM, CPA, EVT, OAX, PET, SMOC, SMOR, SMS, SOC, SON, TAM, YUC. [MTZ].

**Hesperelaea* A. Gray, 1876. 1/1. CAL. [NEA].

*Menodora* Humb. & Bonpl., 1809. 23/20. ALTN, ALTS, BAL, CAL, CGM, CPA, EVT, OAX, SMOC, SMOR, SON, TAM. [MTZ].

**Onagraceae**

*Calylophus* Spach, 1835. 11/10. ALTN, ALTS, SMOC, SMOR, SON, TAM. [NEA, MTZ].

*Camissoniopsis* W.L. Wagner & Hoch, 2007. 14/11. BCA, CAL. [NEA].

*Chylismia* (Torr. & A. Gray) Nutt. ex Raim., 1893. 15/10. BCA, CAL, SON. [NEA].

*Eulobus* Nutt. ex Torr. & A. Gray, 1840. 4/4. BCA, CAL, SON. [NEA].

*Gongylocarpus* Schltdl. & Cham., 1830. 3/3. ALCH, ALTN, ALTS, BAL, BCA, CGM, CPA, EVT, OAX, SMOC, SMS, SOC. [NEO, MTZ].

*Hauya* DC., 1828. 2/2. ALCH, ALTS, BAL, CGM, CPA, EVT, OAX, PET, SMOR, SMS, SOC. [NEO, MTZ].

*Lopezia* Cav., 1791. 31/31. ALCH, ALTN, ALTS, BAL, BCA, CABO, CGM, CPA, EVT, OAX, SMOC, SMOR, SMS, SOC, SON. [NEA, MTZ].

**Megacorax* S. González & W.L. Wagner, 2002. 1/1. ALTN, SMOC. [NEA].

*Tetrapteron* (Munz) W.L. Wagner & Hoch, 2007. 2/1. CAL. [NEA].

**Xylonagra* Donn. Sm. & Rose, 1913. 2/2. BCA. [NEA].

**Opiliaceae**

*Agonandra* Miers, 1862. 10/5. ALCH, ALTN, ALTS, BAL, CGM, CPA, EVT, OAX, PET, SMOC, SMOR, SMS, SOC, TAM, YUC. [NEO, MTZ].

**Orchidaceae**

**Alamania* Lex., 1926. 2/2. ALTS, EVT, OAX, SMOR. [NEA, MTZ].

**Amoana* Leopardi & Carnevali, 2012. 2/2. SMS. [NEA].

*Arpophyllum* Lex., 1825. 5/5. ALCH, BAL, CGM, CPA, EVT, OAX, PET, SMOR, SMS, SOC. [NEO, MTZ].

**Artorima* Dressler & G.E. Pollard, 1971. 1/1. OAX, SMS. [NEA, MTZ].

*Aulosepalum* Garay, 1982. 8/7. ALCH, ALTS, BAL, CGM, CPA, EVT, OAX, SMOC, SMOR, SMS, SOC. [NEO, MTZ].

*Barkeria* Knowles & Westc., 1838. 15/15. ALCH, BAL, CGM, CPA, EVT, OAX, SMOC, SMOR, SMS, SOC. [NEA, MTZ].

*Bletia* Ruiz & Pav., 1794. 33/25. ALCH, ALTN, ALTS, BAL, CABO, CGM, CPA, EVT, OAX, PET, SMOC, SMOR, SMS, SOC, YUC. [MTZ].

*Clowesia* Lindl., 1848. 7/5. ALCH, BAL, CGM, CPA, EVT, SMOC, SMS, SOC. [NEO, MTZ].

*Coelia* Lindl., 1830. 5/5. ALCH, BAL, CGM, CPA, EVT, OAX, SMOR, SOC. [NEO, MTZ].

*Corallorhiza* Gagnebin, 1755. 11/8. ALCH, ALTN, ALTS, CABO, EVT, OAX, SMOC, SMOR, SMS, SOC, SON, TAM. [MTZ].

*Crossoliparis* Marg., 2009. 1/1. ALCH, EVT, SMS. [NEO, MTZ].

*Cryptarrhena* R. Br., 1816. 3/2. CGM, SOC. [NEO].

*Cuitlauzina* Llave, 1825. 7/7. ALCH, BAL, CGM, CPA, EVT, SMOC, SMOR, SMS, SOC. [NEA, MTZ].

*Deiregyne* Schltr., 1920. 19/13. ALCH, ALTN, ALTS, BAL, CPA, EVT, OAX, SMOC, SMOR, SMS, SOC, TAM. [MTZ].

*Dichromanthus* Garay, 1980. 5/5. ALCH, ALTN, ALTS, BAL, CABO, CGM, CPA, EVT, OAX, SMOC, SMOR, SMS, SOC, SON. [NEA, MTZ].

*Dinema* Lindl., 1831. 1/1. ALCH, CGM, CPA, EVT, OAX, PET, SMOR, SOC. [NEO, MTZ].

*Domingoa* Schltr., 1913. 4/3. ALCH, CGM, CPA, EVT, OAX, SMOR, SMS, SOC. [NEO, MTZ].

*Erycina* Lindl., 1853. 7/5. ALCH, BAL, CGM, CPA, EVT, OAX, PET, SMS, SOC, YUC. [NEO, MTZ].

*Funkiella* Schltr., 1920. 7/7. ALCH, ALTN, ALTS, BAL, CGM, CPA, EVT, OAX, PET, SMOC, SMOR, SMS, SOC, SON, YUC. [NEA, MTZ].

*Galeoglossum* A. Rich. & Galeotti, 1845. 3/3. ALCH, EVT, OAX, SMOC, SMOR, SMS. [NEA, MTZ].

*Galeottiella* Schltr., 1920. 2/1. ALCH, ALTS, EVT, OAX, SMOC, SMOR, SMS, SOC. [NEO, MTZ].

*Govenia* Lindl., 1831. 24/16. ALCH, ALTN, ALTS, BAL, CGM, CPA, EVT, OAX, SMOC, SMOR, SMS, SOC. [NEO, MTZ].

*Guarianthe* Dressler & W.E. Higgins, 2003. 4/4. ALCH, BAL, CGM, CPA, EVT, SMOC, SMS, SOC. [MTZ].

**Hagsatera* R. González, 1974. 2/2. BAL, CPA, EVT, SMS. [NEA, MTZ].

*Helleriella* A.D. Hawkes, 1966. 3/2. SMS, SOC. [NEO].

*Hexalectris* Raf., 1825. 10/8. ALTN, ALTS, BAL, CABO, CGM, CPA, EVT, OAX, SMOC, SMOR, SMS, SON, TAM. [NEA, MTZ].

**Hintonella* Ames, 1938. 1/1. EVT, SMS. [NEA, MTZ].

*Ionopsis* Kunth, 1815. 2/2. ALCH, BAL, CGM, CPA, OAX, PET, SMS, SOC, YUC. [NEO, ZTM].

*Jacquiniella* Schltr., 1920. 12/7. ALCH, BAL, CGM, CPA, EVT, OAX, PET, SMOC, SMOR, SMS, SOC. [NEO, MTZ].

*Lacaena* Lindl., 1843. 2/1. ALCH, CGM, SOC. [NEO].

*Laelia* Lindl., 1831. 14/14. ALCH, ALTS, BAL, CGM, CPA, EVT, OAX, PET, SMOC, SMOR, SMS, SOC. [NEO, MTZ].

*Leochilus* Knowles & Westc., 1838. 11/7. ALCH, BAL, CGM, CPA, EVT, OAX, PET, SMOR, SMS, SOC. [MTZ].

*Lyroglossa* Schltr., 1920. 2/1. [NEA].

*Meiracyllium* Rchb. f., 1854. 2/2. ALCH, BAL, CPA, EVT, SMOC, SMS, SOC. [NEO, MTZ].

*Mesadenus* Schltr., 1920. 6/4. ALCH, ALTN, ALTS, BAL, CPA, EVT, OAX, SMOC, SMOR, SMS, SOC. [MTZ].

**Mexipedium* V.A. Albert & M.W. Chase, 1992. 1/1. SOC. [NEA].

**Microepidendrum* Brieger ex W.E. Higgins, 2002. 1/1. SMS. [NEA, MTZ].

*Mormolyca* Fenzl, 1978. 1/1. ALCH, CGM, CPA, PET, SOC. [NEA].

*Nemaconia* Knowles & Westc., 1838. 6/6. ALCH, BAL, CGM, EVT, OAX, PET, SMOR, SMS, SOC. [NEO, MTZ].

*Nidema* Britton & Millsp., 1920. 2/1. ALCH, BAL, CGM, CPA, EVT, OAX, PET, SMOR, SMS, SOC. [NEO, MTZ].

*Oestlundia* W.E. Higgins, 2001. 5/4. ALCH, BAL, CGM, CPA, EVT, OAX, SMOR, SMS, SOC. [NEO, MTZ].

**Physogyne* Garay, 1982. 3/3. BAL, CPA, EVT, SMS. [NEA, MTZ].

*Ponera* Lindl., 1831. 2/2. ALCH, EVT, OAX, SMS. [NEO, MTZ].

*Rhyncholaelia* Schltr., 1918. 2/2. ALCH, CGM, CPA, OAX, PET, SOC, YUC. [NEO].

*Rhynchostele* Rchb., 1852. 19/16. ALCH, BAL, CGM, CPA, EVT, OAX, SMOC, SMOR, SMS, SOC. [NEO, MTZ].

*Rossioglossum* (Schltr.) Garay & G. C. Kenn., 1976. 6/5. ALCH, BAL, CGM, CPA, EVT, SMOC, SMS, SOC. [NEA, MTZ].

*Schiedeella* Schltr., 1920. 20/11. ALCH, ALTN, ALTS, BAL, CABO, EVT, OAX, SMOC, SMOR, SMS, SOC. [MTZ].

*Sotoa* Salazar, 2010. 1/1. ALTN, ALTS. [NEO, MTZ].

**Svenkoeltzia* Burns-Bal., 1989. 1/1. EVT, OAX, SMS. [NEA, MTZ].

*Tamayorkis* Szlach., 1995. 4/3. ALCH, ALTN, ALTS, BAL, EVT, OAX, SMOC, SMOR, SMS, SOC, SON. [NEA, MTZ].

*Wullschlaegelia* Rchb. f., 1863. 2/1. ALCH, CGM. [NEO].

**Orobanchaceae**

*Epifagus* Nutt., 1818. 2/1. SMOR. [NEA, MTZ].

*Escobedia* Ruiz & Pav., 1794. 10/7. ALCH, BAL, CGM, EVT, OAX, SMOC, SMOR, SMS, SOC. [NEO, MTZ].

*Lamourouxia* Kunth, 1817. 25/22. ALCH, ALTN, ALTS, BAL, CGM, CPA, EVT, OAX, SMOC, SMOR, SMS, SOC, SON. [NEO, MTZ].

*Seymeria* Pursh, 1814. 18/16. ALTN, ALTS, BAL, CPA, EVT, OAX, SMOC, SMOR, SMS, SON. [NEA, MTZ].

**Silviella* Pennell, 1928. 2/2. ALTS, OAX. [NEA, MTZ].

**Papaveraceae**

*Argemone* L., 1753. 30/20. ALCH, ALTN, ALTS, BAL, BCA, CABO, CAL, CGM, CPA, EVT, OAX, PET, SMOC, SMOR, SMS, SOC, SON, TAM, YUC. [MTZ].

*Bocconia* L., 1753. 11/8. ALCH, ALTN, ALTS, BAL, CGM, CPA, EVT, OAX, PET, SMOC, SMOR, SMS, SOC. [MTZ].

*Dendromecon* Benth., 1834. 2/1. CAL. [NEA].

*Eschscholzia* Cham., 1820. 12/8. ALTN, ALTS, BCA, CABO, CAL, SON. [MTZ].

**Hunnemannia* Sweet, 1828. 2/2. ALTN, ALTS, OAX, SMOR. [NEA, MTZ].

*Platystemon* Benth., 1835. 1/1. BCA, CAL, SON. [NEA].

*Romneya* Harv., 1845. 2/2. CAL. [NEA].

*Stylomecon* G. Taylor, 1930. 1/1. CAL. [NEA].

**Passifloraceae**

*Erblichia* Seem., 1854. 1/1. ALCH, CGM, CPA, EVT, OAX, PET, SMOC, SMOR, SOC, YUC. [NEA].

**Pentaphylacaceae**

*Symplococarpon* Airy Shaw, 1937. 2/2. ALCH, BAL, CGM, CPA, EVT, OAX, PET, SMOC, SMS, SOC. [NEO, MTZ].

**Petenaeaceae**

*Petenaea* Lundell, 1962. 1/1. ALCH, CGM, PET, SOC. [NEA].

**Petiveriaceae**

*Petiveria* L., 1753. 1/1. ALCH, ALTS, BAL, CABO, CGM, CPA, EVT, OAX, PET, SMOC, SMOR, SMS, SOC, TAM, YUC. [MTZ].

*Rivina* L., 1753. 1/1. ALCH, ALTN, ALTS, BAL, BCA, CABO, CGM, CPA, EVT, OAX, PET, SMOC, SMOR, SMS, SOC, SON, TAM, YUC. [MTZ].

**Phrymaceae**

*Hemichaena* Benth., 1841. 5/5. ALCH, ALTN, ALTS, BAL, CGM, CPA, OAX, SMOR, SMS, SOC. [NEO, MTZ].

*Leucocarpus* D. Don, 1830. 1/1. ALCH, CGM, CPA, EVT, OAX, SMOR, SMS, SOC. [NEO, MTZ].

*Mimulus* L., 1753. 30/20. ALTN, ALTS, BAL, BCA, CABO, CAL, CPA, EVT, SMOC, SMOR, SON. [MTZ].

**Phyllanthaceae**

*Astrocasia* B.L. Rob. & Millsp., 1905. 6/4. ALCH, ALTS, BAL, CGM, CPA, EVT, OAX, PET, SMOR, SMS, SOC, YUC. [NEO, MTZ].

*Phyllanthopsis* (Scheele) Voronts. & Petra Hoffm., 2008. 2/1. ALTN. [NEA].

*Savia* Willd., 1805. 2/1. CGM, CPA, OAX, PET, YUC. [NEO, MTZ].

**Phytolaccaceae**

*Agdestis* Moç. & Sessé ex DC., 1818. 1/1. ALCH, CGM, CPA, EVT, OAX, SMOR. [MTZ].

**Nowickea* J. Martínez & J.A. McDonald, 1989. 2/2. BAL. [NEA, MTZ].

**Picrodendraceae**

*Tetracoccus* Engelm. ex Parry, 1885. 5/4. ALTN, BCA, CABO, CAL. [NEA].

**Plantaginaceae**

*Asarina* Mill., 1757. 2/2. BCA, SON. [NEA].

*Benjaminia* Vell., 1825. 1/1. SOC. [NEO].

*Galvezia* Dombey ex A. Juss., 1789. 4/4. BCA, CABO, SMOC, SON. [NEA, MTZ].

*Keckiella* Straw, 1967. 7/4. BCA, CAL, SON. [NEA].

*Lophospermum* D. Don, 1827. 5/5. ALCH, EVT, OAX, SMOR, SOC. [MTZ].

*Mabrya* Elisens, 1985. 6/5. ALTN, ALTS, CPA, SMOC, SMOR, SON. [NEA, MTZ].

*Maurandya* Ortega, 1797. 7/7. ALCH, ALTN, ALTS, BAL, CGM, EVT, OAX, SMOC, SMOR, SMS, SON, TAM. [MTZ].

*Mohavea* A. Gray, 1856. 2/1. BCA, CAL, SON. [NEA].

*Rhodochiton* Zucc. ex Otto & D. Dietr., 1834. 4/3. OAX, SMS, SOC. [NEO].

*Russelia* Jacq., 1760. 50/43. ALCH, ALTN, ALTS, BAL, BCA, CABO, CGM, CPA, EVT, OAX, PET, SMOC, SMOR, SMS, SOC, YUC. [MTZ].

*Schistophragma* Benth., 1846. 4/4. ALCH, ALTN, ALTS, BAL, BCA, CABO, CGM, CPA, OAX, PET, SMOC, SMOR, SMS, SOC, SON, YUC. [MTZ].

*Uroskinnera* Lindl., 1857. 5/4. ALCH, CGM, CPA, OAX, SOC. [NEA].

**Platanaceae**

*Platanus* L., 1753. 9/7. ALCH, ALTN, ALTS, CAL, CGM, CPA, EVT, OAX, SMOC, SMOR, SOC, SON, TAM. [MTZ].

**Plocospermataceae**

*Plocosperma* Benth., 1876. 1/1. BAL, CPA, OAX. [NEO, MTZ].

**Poaceae**

*Aakia* J.R. Grande, 2014. 1/1. CGM, CPA, PET, SOC. [NEA].

*Achnatherum* P. Beauv., 1812. 14/14. ALTN, ALTS, BAL, CAL, EVT, OAX, SMOC, SMOR, SMS, SON. [MTZ].

*Allolepis* Soderstr. & Decker, 1965. 1/1. ALTN, ALTS. [NEA].

*Anthaenantia* P. Beauv., 1812. 4/2. ALCH, CGM, CPA, EVT, OAX, SMS, SOC. [NEO, MTZ].

**Bealia* Scribn., 1890. 1/1. ALTN. [NEA].

*Blepharoneuron* Nash, 1898. 2/1. ALTN, SMOC. [NEA, MTZ].

*Bouteloua* Lag., 1805. 60/57. ALCH, ALTN, ALTS, BAL, BCA, CABO, CAL, CGM, CPA, EVT, OAX, PET, SMOC, SMOR, SMS, SOC, SON, TAM, YUC. [MTZ].

*Chaboissaea* E. Fourn., 1886. 4/3. ALTN, ALTS, EVT, SMOC. [NEO, MTZ].

*Cottea* Kunth, 1829. 1/1. ALTN, ALTS, OAX, SON. [MTZ].

*Diectomis* P. Beauv., 1812. 1/1. ALCH, ALTS, BAL, CGM, CPA, EVT, PET, SMOC, SMS, SOC, YUC. [MTZ].

*Dinebra* Jacq., 1809. 8/6. ALCH, ALTN, ALTS, BAL, BCA, CABO, CGM, CPA, EVT, OAX, PET, SMOC, SMOR, SMS, SOC, SON, YUC. [MTZ].

*Diplachne* P. Beauv., 1812. 2/2. ALTN, ALTS, BAL, BCA, CABO, CAL, CGM, CPA, EVT, OAX, PET, SMOC, SMOR, SON, YUC. [MTZ].

*Echinolaena* Desv., 1813. 2/2. ALCH, CGM, OAX, SOC. [NEO].

*Erioneuron* Nash, 1903. 3/3. ALTN, ALTS, EVT, OAX, SMOC, SMOR, SON, TAM. [MTZ].

*Gouinia* Fourn., 1886. 11/6. BAL, CABO, CGM, CPA, EVT, OAX, PET, SMOC, SMOR, SMS, YUC. [NEO, MTZ].

*Gynerium* Humb. & Bonpl., 1813. 1/1. CGM, PET, SOC. [NEO].

*Hilaria* Kunth, 1816. 10/8. ALCH, ALTN, ALTS, BAL, BCA, CABO, CGM, CPA, EVT, OAX, SMOC, SMOR, SMS, SON. [NEA, MTZ].

*Hopia* Zuloaga & Morrone, 2007. 1/1. ALTN, ALTS, EVT, OAX, SMOC, SMOR, SON. [NEA, MTZ].

*Ixophorus* Schltdl., 1861. 1/1. ALCH, ALTS, BAL, CGM, CPA, EVT, OAX, SMOC, SMS. [NEO, MTZ].

*Jouvea* Fourn., 1876. 2/2. BCA, CABO, CGM, CPA. [NEO, MTZ].

*Kalinia* H.L. Bell & Columbus, 2012. 1/1. ALTN, ALTS. [NEA, MTZ].

*Lasiacis* (Griseb.) Hitchc., 1910. 16/16. ALCH, ALTN, ALTS, BAL, BCA, CABO, CGM, CPA, EVT, OAX, PET, SMOC, SMOR, SMS, SOC, SON, YUC. [MTZ].

*Limnodea* L.H. Dewey, 1894. 1/1. ALTN. [NEA].

*Louisiella* C.E. Hubb. & J. Léonard, 1952. 2/1. ALTS, YUC. [MTZ].

**Metcalfia* Conert, 1960. 1/1. ALTN, ALTS, OAX, SMOR. [NEA, MTZ].

*Mnesithea* Kunth, 1829. 3/3. ALCH, ALTN, ALTS, BAL, BCA, CABO, CGM, CPA, EVT, OAX, PET, SMOC, SMS, SOC, SON, YUC. [MTZ].

*Morronea* Zuloaga & Scataglini, 2014. 6/6. ALCH, BAL, CGM, CPA, EVT, OAX, PET, SMOR, SMS, SOC, YUC. [MTZ].

*Muhlenbergia* Schreb. ex Gmel., 1791. 155/124. ALCH, ALTN, ALTS, BAL, BCA, CABO, CAL, CGM, CPA, EVT, OAX, PET, SMOC, SMOR, SMS, SOC, SON, TAM, YUC. [MTZ].

*Olmeca* Soderstr., 1981. 5/5. ALCH, CGM, CPA, OAX, SMS, SOC. [NEA].

*Oplismenus* P. Beauv., 1807. 7/5. ALCH, ALTN, ALTS, BAL, BCA, CABO, CGM, CPA, EVT, OAX, PET, SMOC, SMOR, SMS, SOC, SON, TAM, YUC. [MTZ].

*Orthoclada* P. Beauv., 1812. 2/1. CGM, SOC. [NEO].

*Otatea* (McClure & Smith) C.E. Calderón & Soderstr., 1973. 13/11. ALCH, ALTN, ALTS, BAL, CGM, CPA, EVT, OAX, PET, SMOC, SMOR, SMS, SOC, SON. [MTZ].

*Pappophorum* Schreb. ex Vahl, 1794. 7/5. ALTN, ALTS, BAL, CGM, CPA, OAX, SMOR. [MTZ].

*Pascopyrum* Á. Löve, 1980. 1/1. ALTN, ALTS, SMOC. [NEA].

*Peyritschia* E. Fourn., 1886. 7/4. ALCH, ALTN, ALTS, BAL, CABO, CGM, CPA, EVT, OAX, SMOC, SMOR, SMS, SOC, SON. [MTZ].

*Pharus* P. Browne, 1756. 7/4. ALCH, BAL, CGM, CPA, EVT, OAX, PET, SMOC, SMS, SOC. [MTZ].

*Pleuraphis* Torr., 1824. 4/3. ALTN, ALTS, BCA, CAL, SON. [NEA, MTZ].

**Schaffnerella* Nash, 1912. 1/1. ALTS. [NEA].

*Scleropogon* Phil., 1870. 1/1. ALTN, ALTS, EVT, OAX, SMOC, SMOR, SON. [MTZ].

*Setariopsis* Scribn., 1896. 2/2. ALCH, ALTN, ALTS, BAL, CABO, CGM, CPA, EVT, OAX, PET, SMOC, SMOR, SMS, SOC, SON, YUC. [MTZ].

**Sohnsia* Airy Shaw, 1965. 1/1. ALTS. [NEA, MTZ].

*Streptochaeta* Schrad. ex Nees, 1829. 3/2. CGM, OAX. [NEO].

*Streptogyna* P. Beauv., 1812. 2/1. CGM. [NEO].

*Trachypogon* Nees, 1829. 4/2. ALCH, ALTN, ALTS, BAL, CABO, CGM, CPA, EVT, OAX, SMOC, SMOR, SMS, SOC, SON. [MTZ].

*Triniochloa* Hitchc., 1913. 6/5. ALCH, ALTN, ALTS, BAL, CGM, CPA, EVT, OAX, SMOC, SMOR, SMS, SOC. [NEO, MTZ].

*Triplasis* P. Beauv., 1812. 2/1. OAX. [NEA].

*Tripsacum* L., 1759. 14/13. ALCH, ALTN, ALTS, BAL, CABO, CGM, CPA, EVT, OAX, PET, SMOC, SMOR, SMS, SOC, SON, YUC. [MTZ].

*Zea* L., 1753. 7/6. ALCH, ALTS, BAL, CAL, CGM, CPA, EVT, OAX, PET, SMOC, SMOR, SMS, SOC, TAM, YUC. [MTZ].

*Zeugites* P. Browne, 1756. 10/10. ALCH, ALTS, BAL, CGM, CPA, EVT, OAX, SMOC, SMOR, SMS, SOC. [NEO, MTZ].

*Zuloagaea* E. Bess, 2006. 1/1. ALCH, ALTN, ALTS, BAL, CABO, CGM, CPA, EVT, OAX, PET, SMOC, SMOR, SMS, SON. [MTZ].

**Podostemaceae**

*Marathrum* Humb. & Bonpl., 1806. 5/5. BAL, CGM, CPA, OAX, SOC. [NEO, MTZ].

**Noveloa* C.T. Philbrick, 2011. 2/2. BAL, CABO, CPA. [NEA, MTZ].

*Tristicha* Thouars, 1806. 1/1. BAL, CGM, CPA, OAX, SMOR, SMS. [MTZ].

**Polemoniaceae**

**Acanthogilia* A.G. Day & Moran, 1986. 1/1. BCA. [NEA].

*Allophyllum* (Nutt.) V.E. Grant & A.D. Grant, 1955. 5/3. BCA, CAL, SMOC, SON. [NEA].

*Bonplandia* Cav., 1800. 2/1. ALCH, ALTS, BAL, CPA, EVT, SMOC, SMOR, SMS, SOC. [NEO, MTZ].

*Bryantiella* J.M. Porter, 2000. 1/1. BCA. [NEO].

*Cobaea* Cav., 1791. 17/10. ALCH, ALTN, ALTS, BAL, CGM, CPA, EVT, OAX, SMOR, SMS, SOC. [MTZ].

*Dayia* J.M. Porter, 2000. 5/4. ALTN, BCA, CPA, SON. [NEA, MTZ].

*Giliastrum* (Brand) Rydb., 1917. 11/10. ALTN, ALTS, SMOC, SMOR, SON, TAM. [MTZ].

*Ipomopsis* Michx., 1803. 29/15. ALTN, ALTS, CAL, EVT, SMOC, SMOR, SON. [MTZ].

*Langloisia* Greene, 1896. 3/3. CAL, SON. [NEA].

*Linanthus* Benth., 1833. 30/16. ALTN, BCA, CABO, CAL, SMOC, SON. [NEA, MTZ].

*Loeselia* L., 1753. 18/15. ALCH, ALTN, ALTS, BAL, BCA, CABO, CGM, CPA, EVT, OAX, PET, SMOC, SMOR, SMS, SOC, SON, YUC. [MTZ].

**Polygalaceae**

*Hebecarpa* (Chodat) J.R. Abbott, 2011. 19/16. ALCH, ALTN, ALTS, BAL, BCA, CABO, CGM, CPA, EVT, OAX, SMOC, SMOR, SMS, SOC, SON, TAM. [MTZ].

*Rhinotropis* (S.F. Blake) J.R. Abbott, 2011. 17/13. ALCH, ALTN, ALTS, BAL, BCA, CAL, CGM, CPA, OAX, SMOC, SMOR, SOC, SON, TAM. [NEA, MTZ].

**Polygonaceae**

*Antigonon* Endl., 1837. 4/4. ALCH, ALTN, ALTS, BAL, BCA, CABO, CGM, CPA, EVT, OAX, PET, SMOC, SMOR, SMS, SOC, SON, TAM, YUC. [MTZ].

*Centrostegia* A. Gray ex Benth., 1856. 1/1. CAL. [NEA].

*Gymnopodium* Rolfe, 1901. 2/1. CGM, CPA, PET, SOC, YUC. [NEO].

**Harfordia* Greene & Parry, 1886. 3/3. BCA. [NEA].

*Lastarriaea* Remy, 1849. 3/3. BCA. [NEA].

*Nemacaulis* Nutt., 1848. 1/1. BCA, CAL, SON. [NEA].

*Podopterus* Humb. & Bonpl., 1809. 3/3. ALTS, BAL, CGM, CPA, OAX, PET, SOC, YUC. [MTZ].

*Pterostegia* Fisch. & Mey., 1835. 1/1. BCA, CAL. [NEA, MTZ].

**Pontederiaceae**

*Heteranthera* Ruiz & Pav., 1794. 16/9. ALCH, ALTN, ALTS, BAL, CGM, CPA, EVT, OAX, PET, SMOC, SMOR, SMS, SOC, SON, TAM. [MTZ].

**Primulaceae**

*Deherainia* Decne., 1876. 3/3. CGM, CPA, OAX, PET, SOC. [NEO].

*Parathesis* Hook. f., 1876. 108/59. ALCH, BAL, CGM, CPA, EVT, OAX, PET, SMOC, SMOR, SMS, SOC, YUC. [NEO, MTZ].

*Samolus* L., 1753. 14/9. ALCH, ALTN, ALTS, BAL, BCA, CABO, CAL, CGM, CPA, EVT, OAX, PET, SMOC, SMOR, SMS, SOC, SON, TAM, YUC. [MTZ].

*Synardisia* (Mez) Lundell, 1963. 1/1. ALCH, BAL, EVT, SMS, SOC. [NEO, MTZ].

**Resedaceae**

*Forchhammeria* Liebm., 1854. 13/9. ALCH, BAL, BCA, CABO, CGM, CPA, EVT, OAX, PET, SMOR, SMS, SOC, SON, YUC. [NEO, MTZ].

**Rhamnaceae**

*Adolphia* Meisn., 1837. 2/2. ALTN, ALTS, BCA, CAL, EVT, OAX, SMOC, SMOR, SON. [NEA, MTZ].

*Colubrina* Rich. ex Brongn., 1827. 37/19. ALCH, ALTN, ALTS, BAL, BCA, CABO, CGM, CPA, EVT, OAX, PET, SMOC, SMOR, SMS, SOC, SON, TAM, YUC. [MTZ].

*Condalia* Cav., 1799. 18/18. ALTN, ALTS, BAL, BCA, CABO, CGM, CPA, EVT, OAX, SMOC, SMOR, SMS, SOC, SON, TAM. [NEA, MTZ].

*Karwinskia* Zucc., 1832. 10/10. ALCH, ALTN, ALTS, BAL, BCA, CABO, CGM, CPA, EVT, OAX, PET, SMOC, SMOR, SMS, SOC, SON, TAM, YUC. [MTZ].

*Krugiodendron* Urb., 1902. 1/1. ALCH, ALTS, BAL, CGM, CPA, EVT, PET, SMOR, SOC, TAM, YUC. [MTZ].

*Pseudoziziphus* Hauenschild, 2016. 2/1. BCA, CAL. [NEA].

*Ventia* Hauenschild, 2016. 6/5. ALCH, ALTN, ALTS, BCA, CAL, EVT, OAX, SMOC, SMOR, SMS, SOC, SON. [NEA, MTZ].

**Rosaceae**

*Adenostoma* Hook. & Arn., 1832. 3/3. BCA, CAL. [NEA].

*Cercocarpus* Kunth, 1823. 13/13. ALTN, ALTS, BAL, BCA, CAL, CGM, CPA, EVT, OAX, SMOC, SMOR, SMS, SOC, SON. [NEA, MTZ].

*Chamaebatia* Benth., 1849. 2/1. CAL. [NEA].

*Fallugia* Endl., 1840. 1/1. ALTN, ALTS, SMOC, SON. [NEA, MTZ].

*Heteromeles* M. Roem., 1837. 2/2. BCA, CABO, CAL. [NEA].

*Holodiscus* (K. Koch) Maxim., 1879. 9/6. ALCH, ALTN, ALTS, CAL, EVT, OAX, SMOC, SMOR, SMS, SOC, SON. [MTZ].

**Lindleya* Kunth, 1824. 1/1. ALTN, ALTS, OAX, SMOR, SMS. [NEA, MTZ].

*Malacomeles* (Decne.) Decne., 1882. 5/5. ALCH, ALTN, ALTS, BAL, CGM, CPA, EVT, OAX, SMOC, SMOR, SMS, SOC. [NEA, MTZ].

*Purshia* DC., 1817. 7/4. ALTN, ALTS, SMOC, SMOR, SON. [NEA, MTZ].

*Vauquelinia* Correa ex Humb. & Bonpl., 1808. 9/9. ALTN, ALTS, BCA, EVT, OAX, SMOC, SMOR, SMS, SON, TAM. [NEA, MTZ].

**Xerospiraea* Henr., 1985. 1/1. ALTN, ALTS, OAX, SMOR, SMS. [NEA, MTZ].

**Rubiaceae**

*Arachnothryx* Planch., 1849. 90/60. ALCH, BAL, CGM, CPA, EVT, OAX, PET, SMOC, SMOR, SMS, SOC, YUC. [NEO, MTZ].

*Asemnantha* Hook. f., 1873. 1/1. PET, YUC. [NEO].

*Balmea* Martínez, 1942. 1/1. ALCH, BAL, CGM, EVT, SMOC, SMS, SOC. [NEO, MTZ].

*Bellizinca* Borhidi, 2004. 1/1. OAX, SMOR. [NEO, MTZ].

*Blepharidium* Standl., 1918. 1/1. ALCH, CGM, PET, SOC, YUC. [NEO, MTZ].

*Bouvardia* Salisb., 1808. 65/59. ALCH, ALTN, ALTS, BAL, CGM, CPA, EVT, OAX, SMOC, SMOR, SMS, SOC, SON, TAM. [NEA, MTZ].

**Carterella* Terrell, 1987. 1/1. CABO. [NEA].

*Cephalanthus* L., 1753. 6/3. ALCH, ALTN, ALTS, BAL, CGM, CPA, EVT, OAX, PET, SMOC, SMOR, SMS, SOC, SON, TAM. [MTZ].

*Chiococca* P. Browne, 1759. 20/16. ALCH, ALTN, ALTS, BAL, BCA, CABO, CGM, CPA, EVT, OAX, PET, SMOC, SMOR, SMS, SOC, TAM, YUC. [MTZ].

*Chione* DC., 1830. 2/2. ALCH, CGM, CPA, OAX, PET, SMOR, SOC. [NEO, MTZ].

**Cigarrilla* Aiello, 1979. 1/1. ALTS. [NEA, MTZ].

**Cosmocalyx* Standl., 1930. 1/1. CPA, OAX, PET, YUC. [NEA, MTZ].

*Coutaportla* Urb., 1923. 3/2. ALTN, ALTS, BAL, OAX, SMOR, SMS. [NEO, MTZ].

*Crusea* Cham. & Schltdl., 1830. 20/17. ALCH, ALTN, ALTS, BAL, CABO, CGM, CPA, EVT, OAX, PET, SMOC, SMOR, SMS, SOC, SON, TAM, YUC. [MTZ].

**Csapodya* Borhidi, 2004. 3/3. ALCH, SOC. [NEA].

*Deppea* Schltdl. & Cham., 1830. 27/27. ALCH, ALTS, BAL, CGM, CPA, EVT, OAX, SMOC, SMOR, SMS, SOC. [NEO, MTZ].

*Didymaea* Hook. f., 1873. 10/9. ALCH, ALTS, EVT, OAX, SMOC, SMOR, SMS, SOC. [NEA, MTZ].

*Diodella* Small, 1913. 5/3. ALCH, BAL, CGM, CPA, EVT, OAX, SMOC, SMOR. [MTZ].

**Diphragmus* C. Presl, 1845. 2/2. ALCH, BAL, BCA, CABO, CPA, SMOC, SMS, SOC. [NEA, MTZ].

*Donnellyanthus* Borhidi, 2011. 1/1. ALCH, CGM, CPA, SOC. [NEO].

**Edithea* Standl., 1933. 9/9. BAL, CGM, OAX, SMS. [NEA, MTZ].

*Eizia* Standl., 1940. 1/1. SOC. [NEO].

*Glossostipula* Lorence, 1986. 2/2. ALCH, CGM, CPA, EVT, OAX, SMOC, SMOR, SMS, SOC. [NEA, MTZ].

**Habroneuron* Standl., 1927. 1/1. CGM. [NEA].

*Hamelia* Jacq., 1760. 16/10. ALCH, ALTN, ALTS, BAL, CGM, CPA, EVT, OAX, PET, SMOC, SMOR, SMS, SOC, SON, YUC. [MTZ].

*Hemidiodia* K. Schum., 1888. 1/1. ALCH, CGM, CPA, OAX, PET, YUC. [NEO].

*Hintonia* Bullock, 1935. 5/3. ALCH, ALTN, ALTS, BAL, CGM, CPA, EVT, OAX, PET, SMOC, SMS, SOC, SON, YUC. [NEO, MTZ].

*Lorencea* Borhidi, 2003. 1/1. ALCH, CGM, SOC. [NEO, MTZ].

**Martensianthus* Borhidi & Lozada-Pérez, 2011. 7/5. ALCH, OAX, SMS, SOC. [NEA].

**Mexotis* Terrell & H. Rob., 2009. 5/5. ALCH, CGM, CPA, EVT, OAX, SMOR, SMS, SOC. [NEA, MTZ].

*Mitchella* L., 1753. 2/1. ALTS, OAX, SMOR, SMS, TAM. [NEA, MTZ].

**Nernstia* Urb., 1923. 1/1. ALTS. [NEA, MTZ].

*Oldenlandiopsis* Terrell & W.H. Lewis, 1990. 1/1. YUC. [NEO].

**Omiltemia* Standl., 1918. 3/3. SMS. [NEA, MTZ].

*Pinarophyllon* Brandegee, 1914. 2/2. ALCH, CGM, SOC. [NEO].

**Placocarpa* Hook. f., 1873. 1/1. EVT, OAX. [NEA, MTZ].

*Plocaniophyllum* Brandegee, 1914. 1/1. CPA, SOC. [NEA].

**Pseudomiltemia* Borhidi, 2004. 2/2. ALCH, SMS, SOC. [NEA].

*Rachicallis* DC., 1830. 1/1. YUC. [NEO].

*Randia* L., 1753. 109/65. ALCH, ALTN, ALTS, BAL, BCA, CABO, CGM, CPA, EVT, OAX, PET, SMOC, SMOR, SMS, SOC, SON, TAM, YUC. [MTZ].

*Renistipula* Borhidi, 2004. 3/2. ALCH, CGM, OAX, SMOR, SOC. [NEA].

*Rogiera* Planch., 1849. 20/14. ALCH, BAL, CGM, CPA, EVT, OAX, PET, SMOC, SMOR, SMS, SOC. [NEA, MTZ].

*Rovaeanthus* Borhidi, 2004. 2/2. ALCH, CPA, EVT, SMS, SOC. [NEA, MTZ].

*Sommera* Schltdl., 1834. 11/6. ALCH, BAL, CGM, CPA, EVT, OAX, SMOC, SMOR, SMS, SOC. [NEO, MTZ].

**Stenotis* Terrell, 2001. 8/8. ALTS, BCA, CABO. [NEA, MTZ].

*Steyermarkia* Standl., 1940. 1/1. CPA, SOC. [NEO].

*Strumpfia* Jacq., 1760. 1/1. YUC. [NEO].

**Stylosiphonia* Brandegee, 1914. 1/1. SOC. [NEA].

**Syringantha* Standl., 1930. 1/1. ALTS. [NEA, MTZ].

**Rutaceae**

*Amyris* L., 1759. 26/18. ALCH, ALTN, ALTS, BAL, CABO, CGM, CPA, EVT, OAX, PET, SMOC, SMOR, SMS, SOC, SON, TAM, YUC. [MTZ].

*Casimiroa* Llave, 1825. 10/9. ALCH, ALTN, ALTS, BAL, BCA, CABO, CGM, CPA, EVT, OAX, PET, SMOC, SMOR, SMS, SOC, TAM, YUC. [MTZ].

*Choisya* Kunth, 1823. 10/6. ALTN, ALTS, BAL, CGM, EVT, OAX, SMOC, SMOR, SMS. [MTZ].

*Cneoridium* Hook. f., 1862. 1/1. CAL. [NEA].

*Decatropis* Hook. f., 1862. 3/2. ALCH, ALTN, ALTS, CGM, CPA, EVT, SMOR, SOC, TAM. [NEO, MTZ].

*Decazyx* Pittier & S.F. Blake, 1922. 2/1. CGM, SOC. [NEA].

*Ertela* Adans., 1763. 2/1. CPA, EVT, SMS. [NEO, MTZ].

*Esenbeckia* Kunth, 1825. 29/17. ALCH, ALTN, ALTS, BAL, BCA, CABO, CGM, CPA, EVT, OAX, PET, SMOC, SMOR, SMS, SOC, SON, TAM, YUC. [MTZ].

*Megastigma* Hook. f., 1862. 5/5. ALCH, BAL, CPA, OAX, SMS, SOC. [NEA, MTZ].

*Peltostigma* Walp., 1846. 3/3. ALCH, ALTS, CGM, CPA, EVT, OAX, SMOC, SMOR, SMS, SOC. [NEO, MTZ].

**Polyaster* Hook. f., 1862. 1/1. ALTS, OAX. [NEA, MTZ].

*Ptelea* L., 1753. 3/2. ALTN, ALTS, BAL, BCA, CABO, CGM, CPA, EVT, OAX, SMOC, SMOR, SMS, SON, TAM. [MTZ].

*Stauranthus* Liebm., 1853. 2/2. ALCH, OAX, SMOR. [NEA, MTZ].

*Thamnosma* Torr. & Frem., 1845. 7/5. ALTN, ALTS, BCA, CAL, SMOC, SMOR, SON, TAM. [MTZ].

**Salicaceae**

*Hasseltiopsis* Sleumer, 1938. 1/1. ALCH, CGM, EVT, OAX, SMOR, SMS. [NEA, MTZ].

*Neopringlea* S. Watson, 1891. 3/2. ALCH, ALTN, ALTS, BAL, CGM, CPA, EVT, OAX, SMOR, SMS, SOC, TAM. [NEA, MTZ].

*Olmediella* Baill., 1880. 1/1. ALCH, CGM, CPA, SOC. [NEO].

*Pleuranthodendron* L.O. Williams, 1961. 1/1. ALCH, CGM, OAX, PET, SMOR, SOC. [NEO, MTZ].

*Populus* L., 1753. 33/17. ALCH, ALTN, ALTS, BAL, BCA, CABO, CAL, CGM, CPA, EVT, OAX, SMOC, SMOR, SMS, SOC, SON, TAM. [MTZ].

*Prockia* S. Watson, 1887. 6/3. ALCH, ALTS, BAL, CGM, CPA, EVT, OAX, PET, SMOC, SMOR, SMS, SOC, YUC. [NEO, MTZ].

**Santalaceae**

*Arceuthobium* M. Bieb., 1819. 38/20. ALCH, ALTN, ALTS, BAL, CAL, CGM, CPA, EVT, OAX, SMOC, SMOR, SMS, SOC, SON. [MTZ].

*Comandra* Nutt., 1818. 1/1. ALTN, SON. [NEA].

**Sapindaceae**

**Balsas* J. Jiménez Ram. & K. Vega, 2011. 1/1. BAL, SMS. [NEA, MTZ].

*Billia* Peyr., 1858. 2/1. ALCH, CGM, CPA, EVT, OAX, SMOR, SMS, SOC. [NEO].

*Blomia* Miranda, 1953. 1/1. CGM, OAX, PET, SOC, YUC. [NEO].

*Cardiospermum* L., 1753. 10/6. ALCH, ALTN, ALTS, BAL, BCA, CABO, CGM, CPA, EVT, OAX, PET, SMOC, SMOR, SMS, SOC, SON, TAM, YUC. [MTZ].

*Exothea* Macfad., 1837. 4/3. ALCH, ALTS, BAL, CGM, CPA, EVT, OAX, PET, SMOR, SOC, YUC. [MTZ].

*Ungnadia* Endl., 1833. 1/1. ALTN, ALTS, SMOC, SMOR, SON, TAM. [NEA, MTZ].

**Saururaceae**

*Anemopsis* Hook. & Arn., 1841. 1/1. ALTN, ALTS, BCA, CABO, CAL, CPA, SON. [NEA, MTZ].

*Saururus* L., 1753. 2/1. ALTN. [NEA, MTZ].

**Scrophulariaceae**

*Brachystigma* Pennell, 1928. 1/1. SMOC, SON. [NEA, MTZ].

*Emorya* Torr., 1859. 2/2. ALTN, ALTS, SMOR. [NEA].

*Eremogeton* Standl. & L.O. Williams, 1953. 1/1. ALCH, CPA, SOC. [NEO].

*Leucophyllum* Humb. & Bonpl., 1812. 17/17. ALTN, ALTS, EVT, OAX, SMOC, SMOR, TAM. [NEA, MTZ].

*Pseudorontium* (A. Gray) Rothm., 1943. 1/1. BCA, CABO, SON. [NEA, MTZ].

**Setchellanthaceae**

**Setchellanthus* Brandegee, 1909. 1/1. ALTN, ALTS, OAX. [NEA, MTZ].

**Simmondsiaceae**

*Simmondsia* Nutt., 1844. 1/1. BCA, CABO, CAL, SON. [NEA, MTZ].

**Solanaceae**

*Bouchetia* Dunal, 1852. 3/3. ALTN, ALTS, EVT, OAX, SMOC, SMOR, SMS. [MTZ].

*Brachistus* Miers, 1849. 3/3. ALCH, ALTS, BAL, CGM, CPA, EVT, OAX, PET, SMOC, SMOR, SMS, SOC, TAM. [NEO, MTZ].

*Capsicophysalis* Averett & M. Martínez, 2009. 1/1. BAL, CGM, CPA, PET, SOC. [NEA, MTZ].

*Chamaesaracha* (A. Gray) Benth., 1876. 11/11. ALCH, ALTN, ALTS, CGM, CPA, EVT, PET, SMOC, SMOR, SOC, SON, TAM. [NEA, MTZ].

*Datura* L., 1753. 14/11. ALCH, ALTN, ALTS, BAL, BCA, CABO, CAL, CGM, CPA, EVT, OAX, PET, SMOC, SMOR, SMS, SOC, SON, TAM, YUC. [MTZ].

*Hunzikeria* D'Arcy, 1976. 3/2. ALTN, ALTS, TAM. [NEA, MTZ].

*Nectouxia* Kunth, 1818. 2/1. ALTN, ALTS, BAL, CPA, EVT, OAX, SMOC, SMOR, SMS. [NEA, MTZ].

*Physalis* L., 1753. 90/90. ALCH, ALTN, ALTS, BAL, BCA, CABO, CAL, CGM, CPA, EVT, OAX, PET, SMOC, SMOR, SMS, SOC, SON, TAM, YUC. [MTZ].

*Plowmania* Hunz. & Subils, 1986. 1/1. ALCH, SOC. [NEA].

*Schraderanthus* Averett, 2009. 1/1. ALCH, OAX, SMOR, SMS. [MTZ].

*Solandra* Sw., 1787. 10/6. ALCH, ALTS, BAL, CGM, CPA, EVT, OAX, PET, SMOC, SMOR, SMS, SOC, YUC. [NEO, MTZ].

*Tzeltalia* E. Estrada & M. Martínez, 1998. 3/3. ALCH, CGM, CPA, OAX, SOC. [NEO, MTZ].

**Stegnospermataceae**

*Stegnosperma* Benth., 1844. 4/3. BAL, BCA, CABO, CGM, CPA, OAX, SOC, SON. [NEO, MTZ].

**Surianaceae**

*Recchia* Moç. & Sessé ex DC., 1818. 5/4. ALCH, BAL, CGM, CPA, EVT, OAX, SMS, SOC. [NEA, MTZ].

*Suriana* L., 1753. 1/1. CGM, OAX, PET, YUC. [NEO].

**Tetrachondraceae**

*Polypremum* L., 1753. 1/1. ALCH, ALTS, CGM, CPA, SMOC, SMOR, SOC, TAM. [MTZ].

**Thymelaeaceae**

**Dirca* L., 1753. 1/1. SMOR. [NEA, MTZ].

**Ticodendraceae**

*Ticodendron* Gómez-Laurito & L.D. Gómez, 1989. 1/1. ALCH, CGM, OAX, SOC. [NEO].

**Tovariaceae**

*Tovaria* Ruiz & Pav., 1794. 2/1. BAL, CGM, CPA, OAX, SMOR, SMS, SOC. [NEO, MTZ].

**Triuridaceae**

*Lacandonia* E. Martínez & Ramos, 1989. 2/1. CGM. [NEO].

**Ulmaceae**

*Phyllostylon* Capan. ex Benth. & Hook., 1880. 1/1. ALTS, BAL, CGM, CPA, OAX, PET, TAM, YUC. [NEO, MTZ].

**Urticaceae**

*Discocnide* Chew, 1965. 1/1. ALCH, ALTS, BAL, CGM, CPA, EVT, OAX, SMOR, SMS, SOC, YUC. [NEO, MTZ].

*Hesperocnide* Torr., 1857. 2/1. BCA, CAL. [NEA].

*Myriocarpa* Benth., 1844. 12/8. ALCH, ALTS, BAL, CGM, CPA, EVT, OAX, PET, SMOC, SMOR, SMS, SOC. [NEO, MTZ].

**Verbenaceae**

**Burroughsia* Moldenke, 1940. 1/1. BCA. [NEA].

*Phyla* Lour., 1790. 7/7. ALCH, ALTN, ALTS, BAL, CAL, CGM, CPA, EVT, OAX, PET, SMOC, SMOR, SMS, SOC, SON, TAM, YUC. [MTZ].

*Rehdera* Moldenke, 1935. 3/2. CGM, PET, SOC, YUC. [NEO].

*Tamonea* Aubl., 1775. 6/3. ALCH, ALTS, CGM, CPA, OAX, PET, SMOR, SOC, YUC. [NEO, MTZ].

*Xolocotzia* Miranda, 1965. 2/1. CPA, SOC. [NEO].

**Violaceae**

*Ixchelia* H.E. Ballard & Wahlert, 2015. 2/2. ALTN, ALTS, BAL, BCA, CABO, CGM, CPA, EVT, OAX, PET, SMOC, SMOR, SMS, SOC, SON, YUC. [MTZ].

*Orthion* Standl. & Steyerm., 1940. 5/5. ALCH, CGM, OAX, PET, SOC. [NEO].

**Zygophyllaceae**

*Guaiacum* L., 1753. 5/5. ALCH, ALTN, ALTS, BAL, BCA, CABO, CGM, CPA, EVT, OAX, PET, SMOC, SMOR, SMS, SOC, SON, YUC. [MTZ].

*Kallstroemia* Scop., 1777. 18/13. ALCH, ALTN, ALTS, BAL, BCA, CABO, CAL, CGM, CPA, EVT, OAX, PET, SMOC, SMOR, SMS, SOC, SON, TAM, YUC. [MTZ].

**Morkillia* Rose & Painter, 1907. 2/2. ALTS, BAL, OAX. [NEA, MTZ].

**Sericodes* A. Gray, 1852. 1/1. ALTN, ALTS. [NEA].

**Viscainoa* Greene, 1888. 2/2. BCA, CABO, SON. [NEA, MTZ].
